# PPARα and PPARγ are expressed in midbrain dopamine neurons and modulate dopamine- and cannabinoid-mediated behavior in mice

**DOI:** 10.1038/s41380-023-02182-0

**Published:** 2023-07-21

**Authors:** Briana Hempel, Madeline Crissman, Sruti Pari, Benjamin Klein, Guo-Hua Bi, Hannah Alton, Zheng-Xiong Xi

**Affiliations:** 1 Addiction Biology Unit, Molecular Targets and Medications Discovery Branch, National Institute on Drug Abuse, Intramural Research Program, Baltimore, MD, USA.; 2 Medication Development Program, National Institute on Drug Abuse, Intramural Research Program, Baltimore, MD, USA.; 3 Neuropsychopharmacology Section, Molecular Targets and Medications Discovery Branch, National Institute on Drug Abuse, Intramural Research Program, Baltimore, MD, USA.

## Abstract

Peroxisome proliferator-activated receptors (PPARs) are a family of nuclear receptors that regulate gene expression. Δ^9^-tetrahydrocannabinol (Δ^9^-THC) is a PPARγ agonist and some endocannabinoids are natural activators of PPAR*α* and PPARγ. However, little is known regarding their cellular distributions in the brain and functional roles in cannabinoid action. Here, we first used RNAscope in situ hybridization and immunohistochemistry assays to examine the cellular distributions of PPARα and PPARγ expression in the mouse brain. We found that PPARα and PPARγ are expressed in ~70% of midbrain dopamine (DA) neurons. In the amygdala, PPARα is expressed in ~60% of glutamatergic neurons, while PPARγ is expressed in ~60% of GABA neurons. However, no PPARα/γ signal was detected in GABA neurons in the nucleus accumbens. We then used a series of behavioral assays to determine the functional roles of PPARα/γ in the CNS effects of Δ^9^-THC. We found that optogenetic stimulation of midbrain DA neurons was rewarding as assessed by optical intracranial self-stimulation (oICSS) in DAT-cre mice. Δ^9^-THC and a PPARγ (but not PPARα) agonist dose-dependently inhibited oICSS. Pretreatment with PPARα or PPARγ antagonists attenuated the Δ^9^-THC-induced reduction in oICSS and Δ^9^-THC-induced anxiogenic effects. In addition, a PPARγ agonist increased, while PPARα or PPARγ antagonists decreased open-field locomotion. Pretreatment with PPARα or PPARγ antagonists potentiated Δ^9^-THC-induced hypoactivity and catalepsy but failed to alter Δ^9^-THC-induced analgesia, hypothermia and immobility. These findings provide the first anatomical and functional evidence supporting an important role of PPARα/γ in DA-dependent behavior and cannabinoid action.

## INTRODUCTION

In 2020, 49.6 million Americans aged 12 or older used cannabis in the past year and 14.2 million self-reported cannabis use disorder [1]. However, recreational legalization efforts continue to progress; in the last 2 years alone, 5 states have passed legislation allowing non-medical use of marijuana [2]. In this social and legislative climate, a full understanding of cannabis action and the underlying neural mechanisms is critically important. Δ^9^-tetrahydrocannabinol (Δ^9^-THC) is the primary phytocannabinoid within cannabis that is responsible for its subjective effects and many of its therapeutic benefits, which are widely believed to be mediated by activation of cannabinoid type 1 (CB1) and type 2 (CB2) receptors [3–6]. In addition to CB1 and CB2 receptors, Δ^9^-THC and other cannabinoids have high binding activity at other receptor sites such as the G protein-coupled receptor 55 (GPR55), the transient receptor potential cation channel (TRPV1), and the peroxisome proliferator-activated receptor gamma (PPARγ) and possibly alpha (PPARα) [5, 7–9]. Evaluating the non-CB1 and non-CB2 receptor mechanisms underlying cannabinoid action will not only increase our understanding of cannabinoid biology but may also lead to the discovery of new interventions for treating cannabis dependence.

In this context, PPARs are of special interest due to their involvement in a number of CNS functions such as pain [10], reward [11], neuroinflammation [12], and learning and memory [13]. Furthermore, the PPARγ agonist pioglitazone, an FDA-approved medication for the treatment of diabetes in humans, has been shown to be highly effective in reducing voluntary alcohol and opioid consumption and alcohol or nicotine-taking behavior in experimental animals [14–16]. However, the neural mechanisms underlying pioglitazone action are poorly understood.

PPARs are transcription factors within a subfamily of nuclear hormone receptors [17]. They are activated by lipophilic compounds and can bind directly to PPAR response elements, which are selective DNA sequences in target genes [12, 18]. The PPAR family contains three isoforms: PPARα, PPARγ, and PPARβ/δ — each with distinct physiological roles [19]. Recent work has identified interactions between these nuclear receptors and the endocannabinoid system. For instance, the synthetic cannabinoid WIN55,212–2 promotes transcriptional activity at both PPARα and PPARγ, as do the endocannabinoids 2-arachidonoyl-glycerol (2-AG) and anandamide [20–24]. As mentioned above, Δ^9^-THC binds to PPARγ [8, 9], but findings regarding Δ^9^-THC’s affinity to PPARα are inconsistent [9, 20]. One report describes no binding affinity to PPARα [20], while another reveals elevated transcriptional activity at PPARα in the presence of Δ^9^-THC [25]. No prior work has evaluated whether Δ^9^-THC binds to PPARβ/δ.

A small body of literature has emerged in the last two decades investigating the role of PPARs in cannabinoid activity outside of the CNS. For instance, in a neuronal cell culture model of Parkinson’s disease, Δ^9^-THC is neuroprotective and this response is blocked and reinstated by a PPARγ antagonist and agonist, respectively [26]. In addition, the tumor suppressant effects of Δ^9^-THC against liver cancer and its vasorelaxant response in the cardiovascular system are mediated by PPARγ activation [27, 28]. However, no prior work has investigated whether PPARs underlie the CNS effects of cannabinoids and little is known regarding the phenotypes of neurons that express PPARs in the brain.

To address these knowledge gaps, we first examined the cellular distributions of PPARα and PPARγ in multiple types of neurons in the midbrain ventral tegmental area (VTA), nucleus accumbens (NAc), and amygdala using double-staining RNAscope in situ hybridization (ISH) and immunohistochemistry (IHC) assays. Given their major distributions in midbrain dopamine (DA) neurons, we then used pharmacological approaches to manipulate PPARα and PPARγ and transgenic and optogenetic approaches to manipulate VTA DA neurons to determine the functional roles of PPARα and PPARγ in cannabinoid action and DA-dependent behavior.

## MATERIALS AND METHODS

### Subjects

Male and female C57BL/6 J mice (25–35 g; The Jackson Laboratory, Bar Harbor, ME) were utilized throughout the studies. Heterozygous DAT-Cre mice (25–35 g, B6.SJL-*Slc6a3*^*tm1.1(Cre)Bkmn*^/J; stock # 006660) were purchased from the Jackson Laboratory and bred at the National Institute on Drug Abuse (NIDA) Intramural Research Program (IRP) and underwent genotyping by Transnetyx for verification. All subjects were kept on a reverse light cycle (lights off at 7:00 am; on at 7:00 pm) and provided with ad lib food and water. The house room temperature was set to 21–23 °C with 40–50% humidity. Experimental procedures adhered to the *Guide for the Care and Use of Laboratory Animals, 8th edition*. The Animal Care and Use Committee at NIDA approved the study protocol.

### Chemicals

Δ^9^-THC was provided by the NIDA pharmacy (Baltimore, MD). The stock solution was dissolved in ethanol at a concentration of 50 mg/ml. We diluted this solution as needed for experimental use in a 5% cremophor (Sigma-Aldrich, St. Louis, MO) saline solution. PPAR antagonists and agonists including GW9662, GW6471, pioglitazone, and GW7647 were purchased from Cayman Chemical (Ann Arbor, MI). Each compound was dissolved in a mixture of 2% DMSO, 3% tween-80 and 95% saline.

### Experiment 1: RNAscope in situ hybridization

We first performed RNAscope in situ hybridization (ISH) to examine the distribution of PPARα and PPARγ mRNA in the mesolimbic DA system and amygdala - — regions associated with the affective properties of cannabinoids. In the VTA, we examined PPARα (*PPARA*) and PPARγ (*PPARG*) mRNA expression in GABAergic (*GAD1*^+^), glutamatergic (*Slc17a6*^+^) and dopaminergic (*TH*^+^) neurons. In the NAc, we focused on PPAR expression in GABAergic (*GAD1*^+^) neurons, whereas in the amygdala, we looked at their expression patterns in GABAergic (*GAD1*^+^) and glutamatergic (*Slc17a6*^+^) neurons. The complete RNAscope procedures are described in Supplementary Information.

### Experiment 2: Immunofluorescence

RNAscope ISH assays detected weak PPARα and PPARγ mRNA signals. To better examine the expression of PPAR receptor proteins on different cell types in the VTA (GABA, glutamate, and TH), NAc (GABA), and amygdala (GABA & glutamate), we used double label immunostaining. The complete immunofluorescence procedures are described in Supplementary Information.

### Experiment 3: Optical intracranial self-stimulation

In Experiment 2, we found that PPARα and PPARγ are highly expressed in midbrain DA neurons. To understand the functional role of these receptors, we next examined how pharmacological manipulation of PPARα and PPARγ altered DA-dependent behavior in the presence or absence of Δ^9^-THC action in an oICSS paradigm. The complete oICSS procedures are described in Supplementary Information.

### Experiment 4: Conditioned place preference or aversion (CPP/CPA)

We then examined whether pretreatment with PPARα or PPARγ antagonists altered the aversive subjective effects of Δ^9^-THC using the CPP test. The complete CPP/CPA procedures are described in Supplementary Information.

### Experiment 5: Elevated plus maze

Next, we considered the role of PPARα and PPARγ in Δ^9^-THC-induced anxiety in the elevated plus maze (EPM). The complete EPM procedures are described in Supplementary Information.

### Experiment 6: Open-field locomotion

In this experiment, we first examined whether PPAR agonists or antagonists alter open-field locomotion by themselves, and then examined whether pretreatment with PPAR antagonists block Δ^9^-THC-induced hypoactivity. The complete locomotor test procedures are described in Supplementary Information.

### Experiment 7: Δ^9^-THC-induced tetrad

Lastly, we looked at whether PPARα and PPARγ mediate the classical tetrad effects produced by high doses (10, 30 mg/kg) of Δ^9^-THC. The complete tetrad experimental procedures are described in Supplementary Information.

### Statistical analyses

All data are presented as means ± SEM. One-way or two-way repeated-measures (RM) analysis of variance (ANOVA) were used to evaluate the effects of testing compounds (CB1, CB2, or PPAR agonists or antagonists) alone or their pretreatment on Δ^9^-THC-induced changes in oICSS, CPP/CPA, anxiety, open-field locomotion, and tetrad effects. oICSS and tetrad data were also analyzed based on changes in the area under the curve (ΔAUC) to better visualize group differences. Data were converted to ΔAUC by summating the difference between each time point after drug injection and a baseline value before the injection. The post-hoc group comparisons were conducted only if the ANOVA *F* value achieved *p* < 0.05. The value of *p* < 0.05 was used to indicate statistically significant differences among or between groups.

Animal group sizes were chosen based on a power analysis (*n* ≥ 8 per group) and extensive previous experience with the animal models used. The group size is the number of independent values (individual animals), and statistical analysis was done using these independent values. No data points were excluded from the analysis in any experiment. The investigators were blinded to the group allocation during the experiments and when assessing the outcome. To validate the use of parametric statistics, we performed a Shapiro Wilk Test for data normality evaluation and Levene’s test for homogeneity for between-subject ANOVA. Estimation statistics were used when necessary (when data were not normally distributed (www.estimationstats.com).

## RESULTS

### Cellular distributions of PPARα and PPARγ in the VTA, NAc, amygdala

**Fig. 1:**
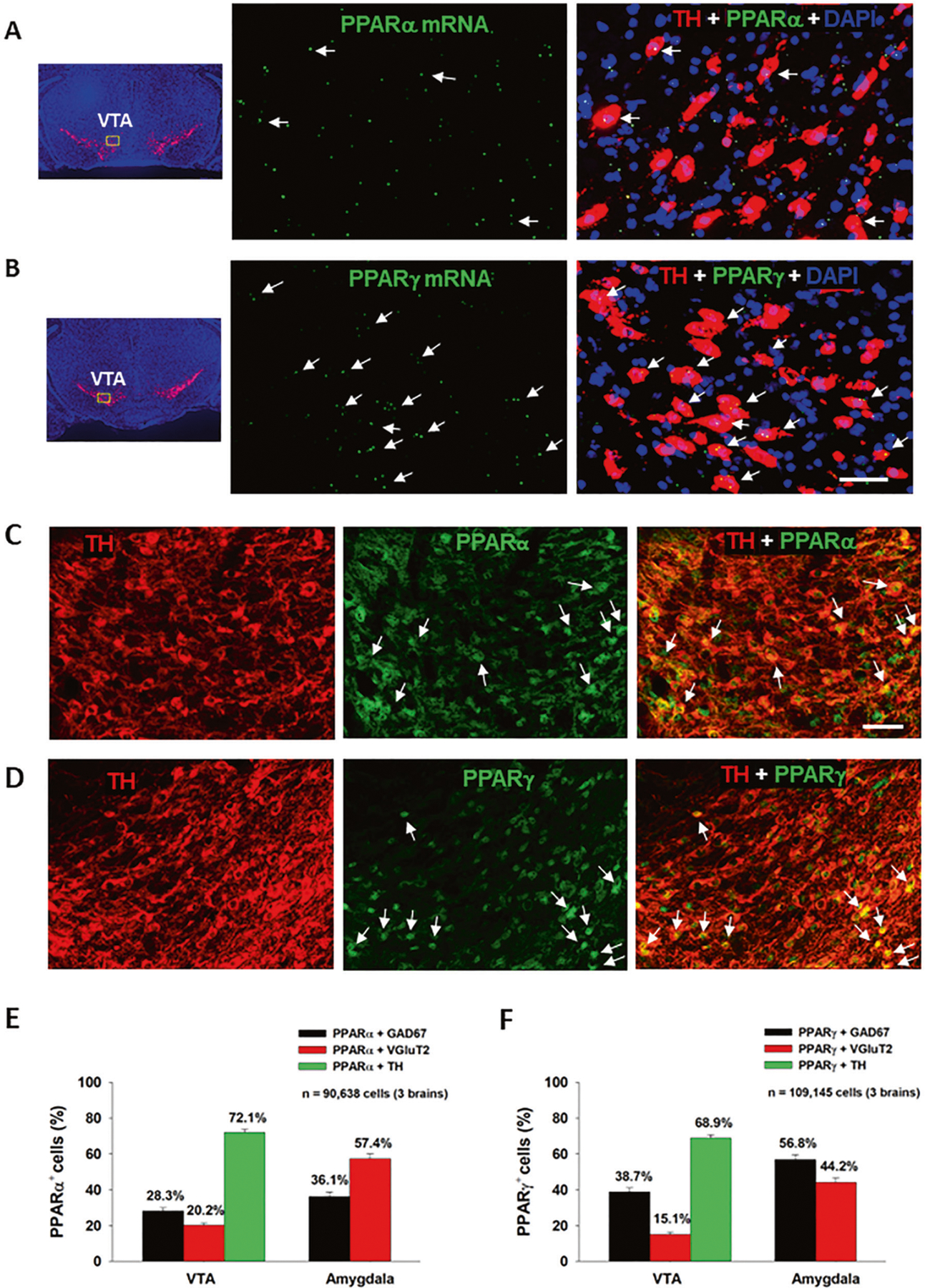
PPARα and PPARγ RNAscope ISH and immunostaining results. **A/B** Representative RNAscope ISH, illustrating low densities of PPARα (**A**) and PPARγ (**B**) mRNA signals detected in TH^+^ DA neurons in the VTA. **C/D** Representative images indicating that PPARα- or PPARγ-immunostaining was co-localized with TH-immunostaining in VTA DA neurons. **E/F** The cell counting data indicate that PPARα and PPARγ are expressed in ~70% of DA neurons in the VTA and in 40–60% of GABA or glutamate neurons in the Amygdala. The scale bar indicates 50 μm. Each bar illustrates the average percentage of cells co-expressing PPARα or PPARγ with one neuronal marker (TH, GAD67 or VGluT2) out of the total number of DA, glutamate or GABA neurons. *N* = 3 brains with 5–6 slices selected from each brain and 2–4 images taken per region/slice. (see Figs. S1–S6 for PPARα or PPARγ mRNA or immunostaining in other types of neurons in the VTA, NAc and amygdala).

We first examined the expression of PPARα and PPARγ in different neuronal phenotypes in the mesolimbic DA system and amygdala, which are critical brain regions involved in cannabinoid action [5]. Figure 1 (A, B) highlights a representative image of PPAR mRNA staining, illustrating that PPARα and PPARγ mRNA are detected in VTA DA neurons. Notably, more DA neurons displayed TH and PPARγ transcript colocalization than DA neurons showing TH and PPARα transcript colocalization (Fig. 1; Fig. S1). PPARα and PPARγ mRNA was also detected in GABA and glutamate neurons in the VTA, NAc and amygdala (Figs. S2, S3). However, in these cell types, PPARα and PPARγ mRNA expression levels were low and some were observed outside of DAPI-labeled nuclei, complicating cell counting analyses. As such, cell counting was not attempted on these data.

The low PPARα and PPARγ mRNA expression levels observed in DA, GABA and glutamate neurons were unexpected given previous work demonstrating a strong neuronal signal using immunofluorescent assays [29]. To address this discrepancy, we utilized double-label IHC to measure protein expression of PPARα and PPARγ in the predominant cell types within the regions of interest. We detected strong PPARα and PPARγ immunostaining in TH^+^ DA neurons in the VTA (Fig. 1C, D) as well as in GAD67^+^ GABA neurons and VgluT2^+^ glutamate neurons in the VTA and amygdala (Figs. S4, S5). In the NAc, no PPAR immunostaining overlapped with GAD67^+^ GABA neurons (Fig. S6). Surprisingly, PPARα and PPARγ immunostaining was detected mainly in astrocyte-like cells in the NAc, suggesting that these may be glial receptors. Quantitative cell counting assays revealed that PPARα and PPARγ are expressed in ~70% of DA neurons, ~30% of GABA neurons and ~20% of glutamate neurons in the VTA (Fig. 1E, F). In the amygdala, PPARα is found in ~60% of glutamate neurons and ~40% of GABA neurons, while PPARγ is expressed in ~60% of GABA neurons and ~40% of glutamate neurons. In the NAc, PPARα/γ and GAD67 co-expression was negligible, so no quantification was performed.

### PPARα/γ modulate DA-dependent oICSS and Δ^9^-THC action in oICSS

We have recently reported that optogenetic stimulation of VTA DA neurons is rewarding as assessed by optical ICSS (oICSS) and real-time place preference [30, 31] and this effect is dose-dependently attenuated by cannabinoids such as Δ^9^-THC, WIN55212,2 or AM-2201 [32]. However, the receptor mechanisms underlying cannabinoid reward-attenuation in oICSS are unclear. Given that Δ^9^-THC is also a PPARγ agonist (EC_50_ = 0.3 μM) [8, 33] and other cannabinoids have binding affinity to PPARα and PPARγ [9], we first examined whether PPAR agonists produce similar effects as Δ^9^-THC and whether pretreatment with PPAR antagonists would block Δ^9^-THC-induced changes in oICSS in transgenic DAT-Cre mice.

**Fig. 2:**
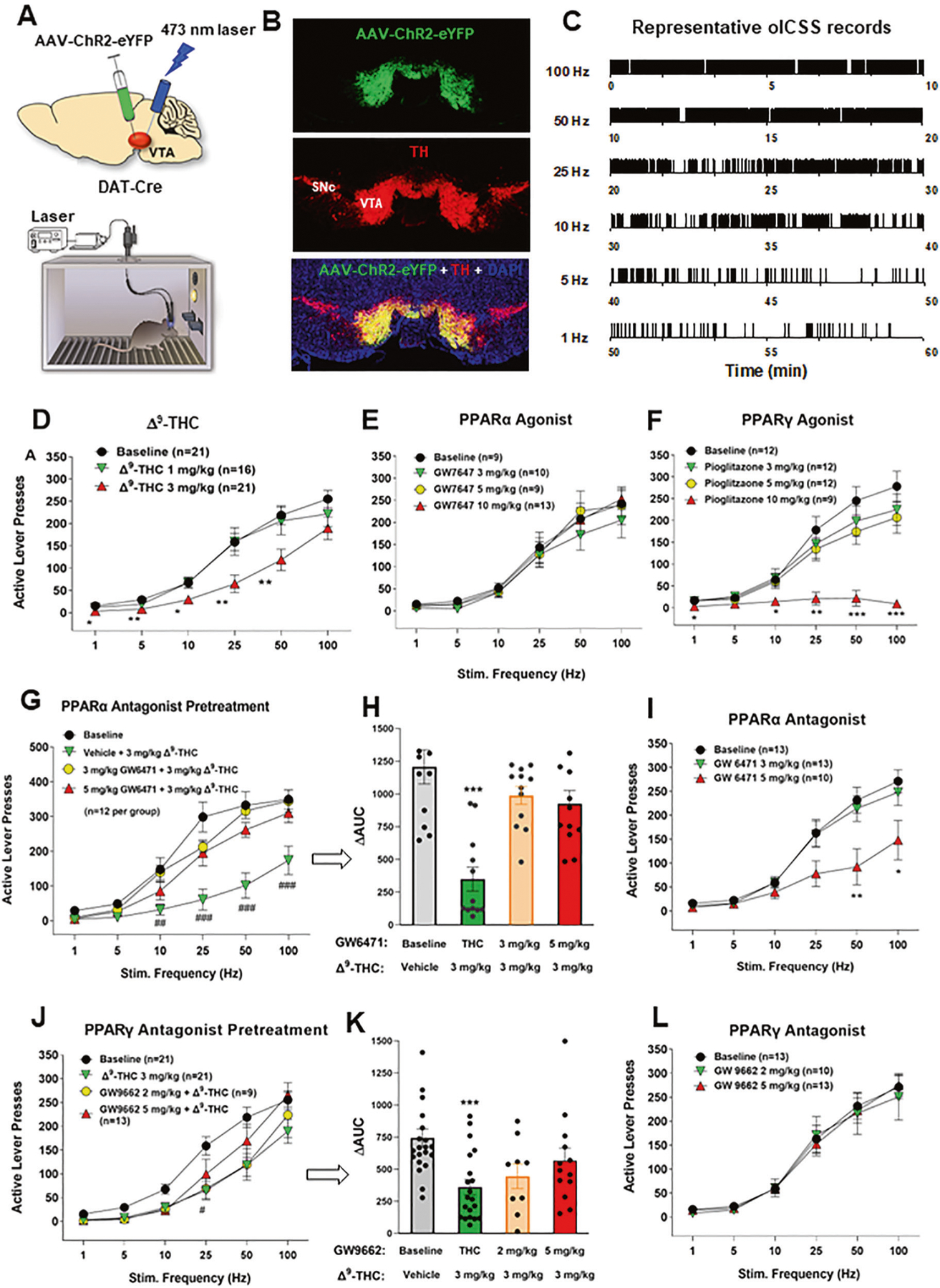
Effects of Δ^9^-THC and/or PPAR agonists and antagonists on optical brain-stimulation reward (oICSS) in DAT-Cre mice. **A** Diagrams showing the general experimental methods. The AAV-ChR2-eYFP vectors were microinjected bilaterally into the midbrain VTA and two optical fibers were targeted to the VTA. Mice were placed in oICSS chambers and trained to press an active lever to obtain laser stimulation reward. **B** Representative images showing AAV-ChR2-eYFP expression in TH^+^ DA neurons in the VTA. **C** Representative lever responding to different frequencies of laser stimulation in a single session from a single mouse. **D** Stimulation–response curve of lever responding over different frequencies of laser stimulation. Δ^9^-THC (1, 3 mg/kg, intraperitoneal, i.p.) dose-dependently shifted the oICSS curve downward compared with the vehicle (baseline) control group. **E/F** PPARγ agonism (by pioglitazone) produced a similar inhibitory effect on oICSS as Δ^9^-THC, while PPARα agonism (by GW7674) failed to alter basal oICSS. **G/H** Pretreatment with GW6471 (a selective PPARα antagonist) dose-dependently attenuated Δ^9^-THC-induced reduction in oICSS. **I** GW6471 dose-dependently decreased oICSS response. **J/K** Pretreatment with GW9662 (a selective PPARγ antagonist) attenuated Δ^9^-THC-induced reduction in oICSS. **L** GW9662 alone failed to alter oICSS. *p < 0.05, **p < 0.01, ***p < 0.001 relative to baseline.

Figure 2 shows the experimental results, indicating that bilateral stimulation of VTA DA neurons maintains robust oICSS behavior in a stimulation frequency-dependent manner (Fig. 2A–C), which is dose-dependently inhibited by systemic administration of Δ^9^-THC (Fig. 2D) or pioglitazone (a PPARγ agonist, Fig. 2F), but not by GW7647 (a selective PPARα agonist, Fig. 2E). A two-way RM ANOVA revealed a significant Δ^9^-THC treatment main effect (Fig. 2D, F_2,49_ = 5.19, *p* < 0.01) and pioglitazone treatment main effect (Fig. 2F, F_3,41_ = 8.15, *p* < 0.001), but a non-significant effect with GW7647 (Fig. 2E, F_3,37_ = 0.44, *p* > 0.05). More detailed statistical analysis results are shown in supplementary Table 1. This finding that a PPARγ, but not PPARα, agonist produces a Δ^9^-THC-like effect in oICSS suggests that Δ^9^-THC may inhibit brain-stimulation reward in part by activation of PPARγ.

To test this hypothesis, we then determined whether a selective PPARα or PPARγ antagonist alters Δ^9^-THC-induced changes in oICSS. We found that pretreatment with GW6471 (a selective PPARα antagonist) significantly attenuated Δ^9^-THC-induced reduction in oICSS at both doses (Fig. 2G, H). A two-way RM ANOVA revealed a significant GW6471 treatment main effect (Fig. 2G, F_3,33_ = 12.87, *p* < 0.001) and treatment X frequency interaction (F_15,165_ = 6.89, *p* < 0.001). Analyzing the changes in the area under curve (ΔAUC) values for the data shown in Fig. 2G also revealed a significant GW6471 pretreatment main effect (Fig. 2H, one-way ANOVA, F_3,33_ = 12.87, *p* < 0.001). Unexpectedly, GW6471 itself produced a dose-dependent reduction in oICSS (Fig. 2I, F_2,33_ = 4.58, *p* < 0.05) whereas the PPARα agonist GW7647 failed to alter oICSS (Fig. 2E), suggesting that PPARα may tonically modulate the mesolimbic DA system under physiological conditions. Thus, the antagonist GW6471 may produce a reduction in oICSS by blockade of endogenous ligand binding to PPARα, while the agonist GW7647 may not work due to a ceiling effect caused by endogenous ligand binding. In addition, PPARα is a transcription factor. Thus, it is likely that PPARα modulates cellular responses in different directions when it is activated or inhibited.

**Fig. 3:**
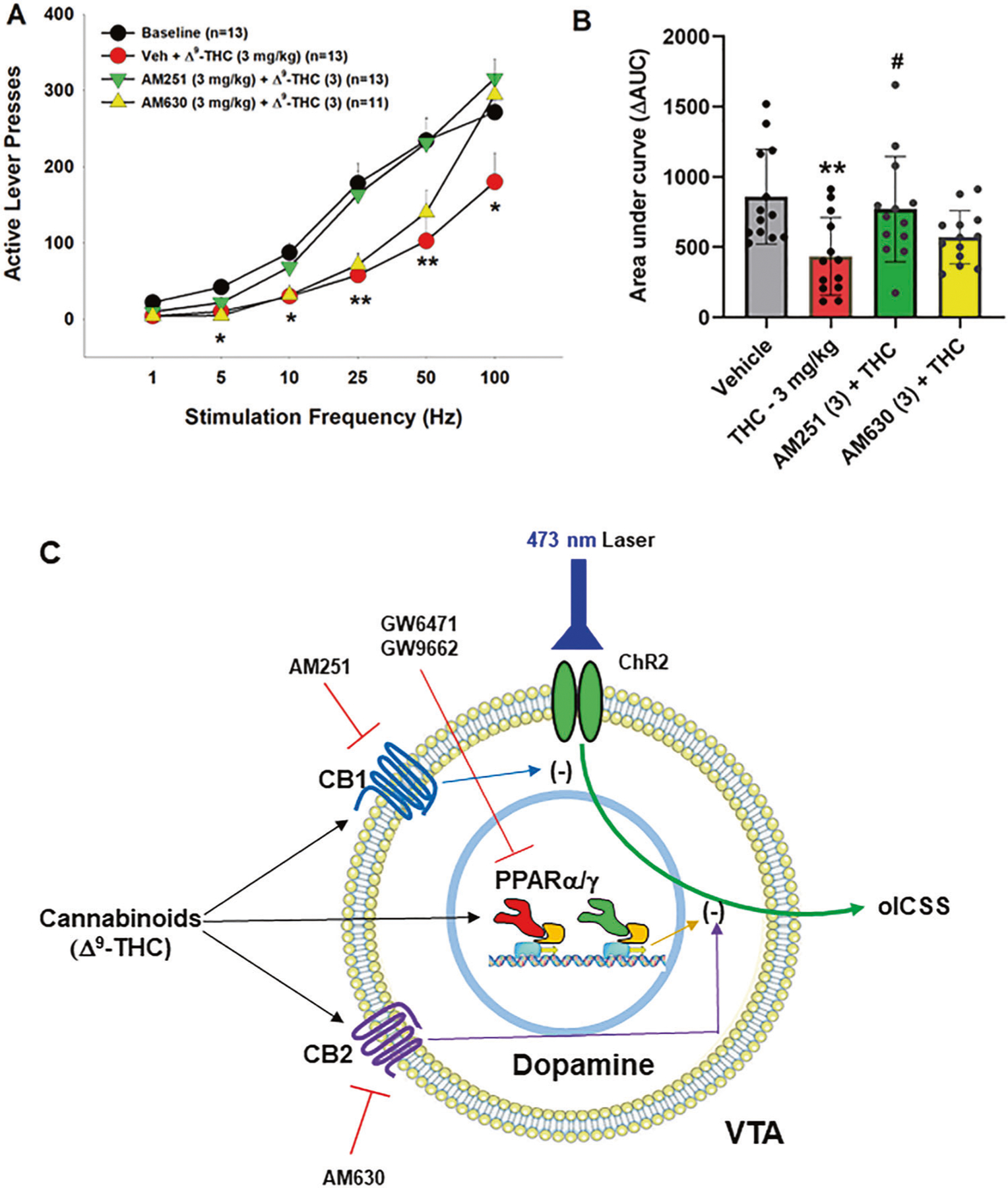
Effects of CB1 and CB2 receptor antagonists on Δ^9^-THC-induced changes in oICSS in DAT-cre mice. **A** The stimulation-rate response curves showing that 3 mg/kg Δ^9^-THC significantly decreased oICSS, which was blocked by AM251 and partially reduced by AM630. **B** The ΔAUC data from the data in (**A**), illustrating that the reduction in oICSS by Δ^9^-THC was blocked by AM251 and partially reduced by AM630. **C** A summary diagram showing how Δ^9^-THC modulates oICSS and how CB1, CB2 and PPAR antagonists block Δ^9^-THC action in oICSS. **p < 0.01, ***p < 0.001, relative to baseline. ^#^*p* < 0.05, relative to ^Δ9^-THC group.

Next, animals were pretreated with a PPARγ antagonist (GW9662). We found that GW9662 pretreatment also attenuated Δ^9^-THC-induced reduction in oICSS in a dose-dependent manner (Fig. 2J, K). Two-way RM ANOVAs over time (stimulation frequency) revealed a statistically significant GW9662 treatment main effect (Fig. 2J, F_3,60_ = 3.83, *p* < 0.05) and treatment X frequency interaction (F_15,300_ = 2.64, *p* < 0.001). Analyzing the changes in the area under curve (ΔAUC) values for the data shown in Fig. 3J also revealed a significant GW9662 pretreatment main effect (Fig. 2K, one-way ANOVA, F_2,54_ = 8.26, *p* < 0.001). Figure 2L shows that administration of GW9662 alone failed to alter oICSS (F_2,33_ = 0.04, *p* = 0.96). More detailed statistical analysis results are shown in the supplementary Table 1. These findings provide the first behavioral evidence indicating that PPARα and PPARγ receptor mechanisms at least in part underlie Δ^9^-THC-induced reward attenuation.

We have previously reported that both CB1 and CB2 receptors are expressed in midbrain DA neurons and glutamate neurons [34–37], which have been thought to play an important role in cannabinoid action [5, 38, 39]. To provide a point of comparison for our PPAR findings, we examined the effects of AM251 (a selective CB1R antagonist) and AM630 (a selective CB2R antagonist) on Δ^9^-THC-induced changes in oICSS. Figure 3 shows that AM251 pretreatment almost completely blocked Δ^9^-THC suppression of oICSS (Fig. 3B, F_3,34_ = 5.76, *p* < 0.01), while AM630 partially reduced Δ^9^-THC activity. This data suggests that CB1R (and CB2R to a lesser extent) are involved in Δ^9^-THC’s aversive effects (Fig. 3C).

### Effects of PPAR antagonists on Δ^9^-THC-induced place aversions

Next, we examined whether pretreatment with PPAR antagonists is able to block Δ^9^-THC-induced conditioned place aversion (CPA) (Fig. S7). Figure S7(B, C) shows that pretreatment with either the PPARα antagonist (GW6471) or PPARγ antagonist (GW9662) failed to alter Δ^9^-THC-induced CPA, suggesting that PPARs are not critically involved in Δ^9^-THC-induced place aversion. This is consistent with our previous reports that CB1 and CB2 receptor mechanisms underlie the rewarding and aversive effects [40, 41]. A two-way RM ANOVA on CPP scores in subjects administered Δ^9^-THC detected a significant main effect of Test (cocaine CPP) (Fig. S7B, F_1,21_ = 13.74, *p* < 0.01), but not GW6471 dose (F_2,21_ = 0.06, *p* = 0.95) or the interaction between these factors (F_2,21_ = 0.007, *p* = 0.99). An identical analysis on CPP scores in subjects administered a PPARγ inhibitor showed a main effect of Test (Fig. S7C, F_1,21_ = 16.7, *p* < 0.001), but no GW9662 dose effect (F_2,21_ = 0.60, *p* = 0.56) or interaction (F_2,21_ = 0.09, *p* = 0.91).

We also examined the effects of the PPAR antagonists alone in CPP. We found that the PPARα antagonist GW6471 (Fig. S7D, F_2,21_ = 1.21, *p* = 0.32) failed to produce either CPP or CPA, while the PPARγ antagonist GW9662, at a low dose (2 mg/kg), produced significant place aversion in the absence of Δ^9^-THC (Fig. S7E, F_1,21_ = 8.95, *p* < 0.01), suggesting that PPARγ tonically modulates brain reward function under physiological conditions.

### Blockade of PPARs attenuates Δ^9^-THC-induced anxiety

**Fig. 4:**
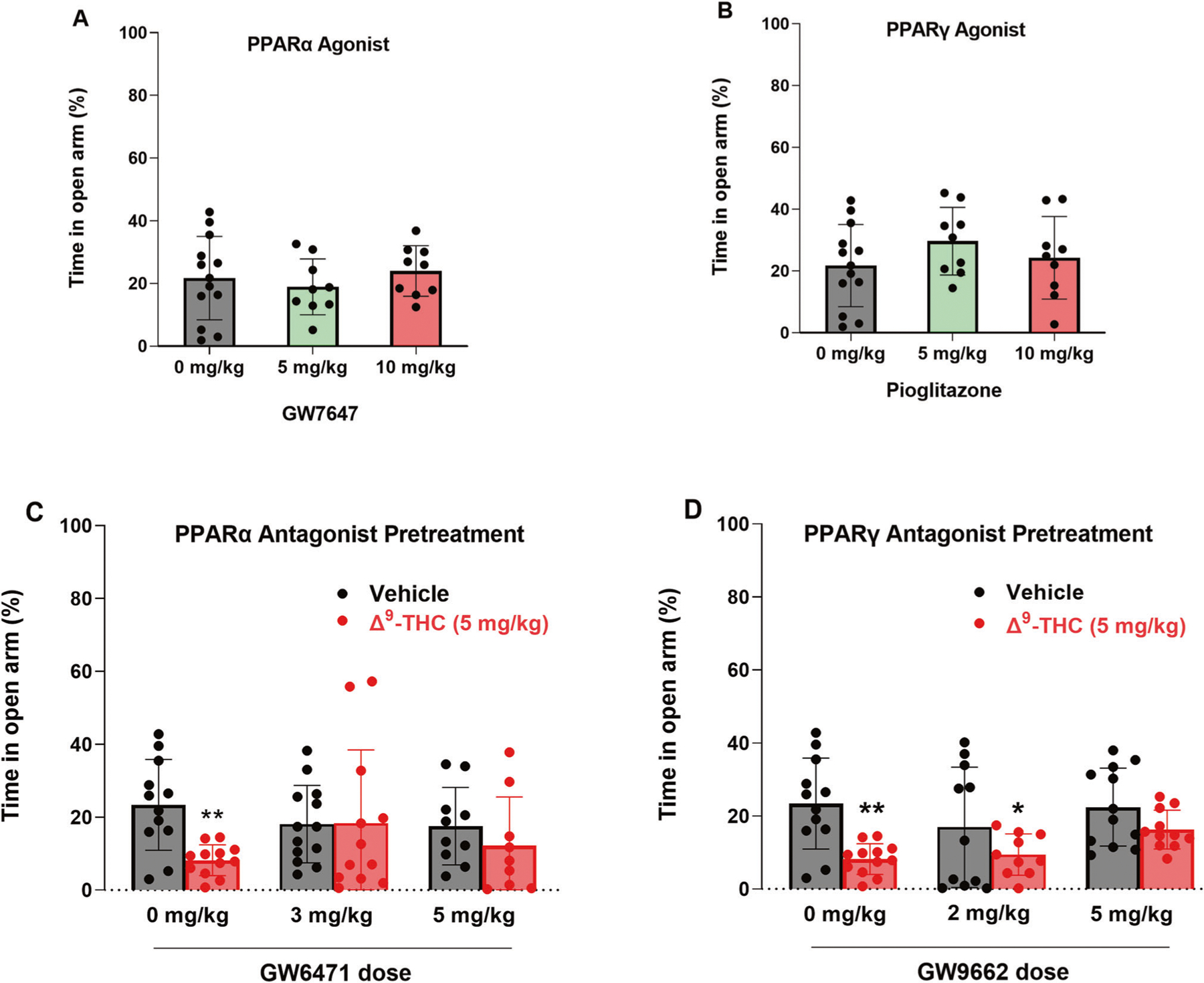
Effects of PPARα and PPARγ antagonists on Δ^9^-THC-induced anxiety in the elevated plus maze test. **A/B** PPARα (GW7647) or PPARγ (pioglitazone) agonism produced neither anxiety nor anxiety relief. **C/D** Pretreatment with PPARα (GW6471) or PPARγ (GW9662) antagonist attenuated Δ^9^-THC-induced anxiety. *p < 0.05, **p < 0.01, relative to vehicle. *n* = 9–13/group.

In addition to VTA DA neurons, PPARα and PPARγ are also expressed in ~60% of GABA and glutamate neurons in the amygdala, a critical brain region involved in affective behavior. Therefore, we further examined the functional roles of PPARs in cannabinoid-induced anxiety (Fig. 4). We first examined the effects of PPAR agonists in an elevated plus maze (EPM) test. We found that systemic administration of PPARα agonist (Fig. 4A, F_2,27_ = 0.67, *p* = 0.52) or PPARγ agonist alone (Fig. 4B, F_2,27_ = 0.73, *p* = 0.49) produced neither an anxiolytic nor anxiogenic response, as assessed by the times the animals spent on the open arm or closed arm of the EPM, respectively. However, pretreatment with either PPARα or PPARγ antagonist significantly attenuated Δ^9^-THC-induced anxiogenic effects (Fig. 4C, D), while PPARα or PPARγ antagonists alone failed to produce anxiogenic or anxiolytic effects (Fig. 4C, D, vehicle groups). These data suggest that PPAR mechanisms are critically involved in the anxiogenic effects of Δ^9^-THC. A two-way ANOVA on percent time in the open arm of the EPM showed a main effect of Δ^9^-THC dose (Fig. 4C, F_1,62_ = 4.706, *p* < 0.05), but not GW6471 dose (F_2,62_ = 0.41, *p* = 0.66) or the interaction between these factors (F_2,62_ = 2.26, *p* = 0.11). Post hoc comparisons revealed that Δ^9^-THC-induced anxiety is statistically significant in the vehicle (0 mg/kg GW6471) control group. However, in subjects pretreated with 3 or 5 mg/kg GW6471, Δ^9^-THC did not produce significant anxiogenic effects relative to vehicle control group (Fig. 4C). Another two-way ANOVA on Δ^9^-THC-induced anxiety produced a main effect of Δ^9^-THC dose (Fig. 4D, F_1,62_ = 18.93, *p* < 0.001), but not GW9662 dose (F_2,62_ = 1.25, *p* = 0.29) or the interaction term (F_2,62_ = 0.68, *p* = 0.51). Post hoc comparisons showed that subjects administered Δ^9^-THC by itself or in conjunction with 2 mg/kg GW9662 were more anxious relative to controls whereas in the group given 5 mg/kg GW9662, Δ^9^-THC did not produce significant anxiogenic effects compared to the vehicle controls (Fig. 4D).

### Effects of Δ^9^-THC and PPAR antagonists on locomotor activity

**Fig. 5:**
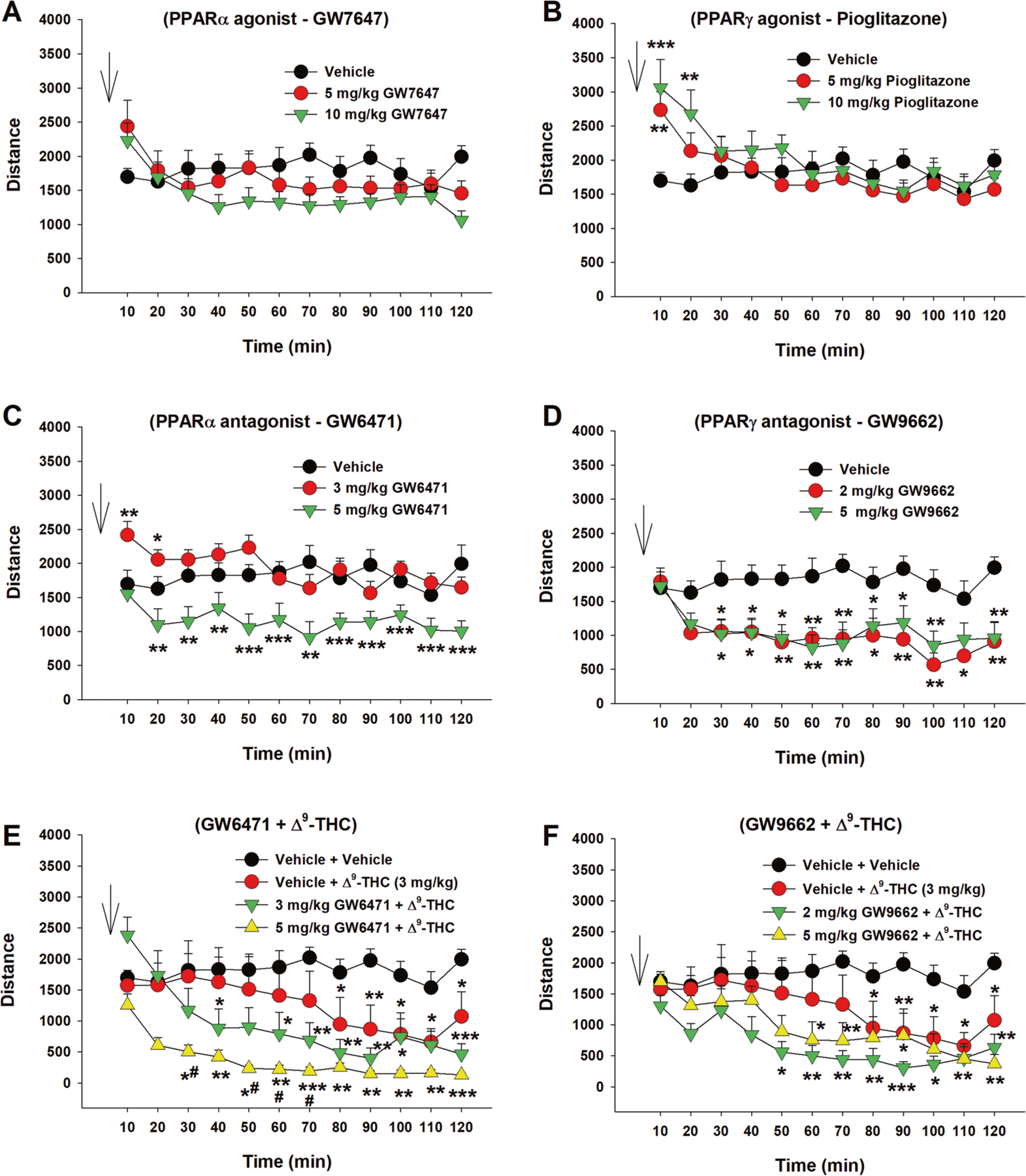
Effects of Δ^9^-THC and/or PPAR agonists or antagonists on open-field locomotion. **A/B** Systemic administration of the PPARα agonist GW7647 failed to alter open-field locomotion (**A**), while the PPARγ agonist pioglitazone produced a transient increase in locomotion (**B**). **C/D** Systemic administration of PPARα antagonist GW6471 (**C**) or PPARγ antagonist GW9662 (**D**) alone dose-dependently inhibited open-field locomotion. **E/F** Pretreatment with GW6471 enhanced Δ^9^-THC-induced reduction in locomotor activity (**E**), while GW9662 pretreatment did not significantly alter Δ^9^-THC action in locomotion (**F**). *n* = 8/group. *p < 0.05, **p < 0.01, ***p < 0.001, compared to the vehicle group. ^#^ *p* < 0.05, compared to the (Vehicle + Δ^9^-THC) group (**E**).

We then examined the effects of Δ^9^-THC with or without PPAR ligands on open-field locomotion (Fig. 5). Systemic administration of a selective PPARα agonist (GW7647) failed to alter locomotor activity (Fig. 5A, F_2,21_ = 0.46, *p* > 0.05), while a selective PPARγ agonist (pioglitazone) produced a significant increase in locomotion, an effect that lasted for about 20 min. A two-way RM ANOVA did not reveal a significant pioglitazone treatment main effect (Fig. 5B, F_2,21_ = 0.44, *p* = 0.65), but revealed a significant treatment × time interaction (F_22,231_ = 5.36, *p* < 0.001). Post hoc group comparisons revealed a significant increase in locomotion at 10 and 20 min after pioglitazone administration compared to the vehicle control group (Fig. 5B). In contrast, systemic administration of PPAR antagonists produced a significant reduction in open-field locomotion (Fig. 5C, D). A two-way RM ANOVA reveal a significant GW6471 treatment main effect (Fig. 5C, F_2,21_ = 17.39, *p* < 0.001) and a significant GW9662 treatment main effect (Fig. 5D, F_2,14_ = 5.67, *p* < 0.01). More detailed statistical results are shown in the supplementary Table 2. These findings suggest that PPARγ tonically modulates basal locomotor behavior under physiological conditions.

We then observed the effects of PPAR antagonist pretreatment on Δ^9^-THC-induced changes in locomotion. We found that systemic administration of 3 mg/kg Δ^9^-THC produced a significant reduction in locomotion (Fig. 5E, F), consistent with our previous finding [42]. However, pretreatment with a selective PPARα antagonist (GW6471) enhanced Δ^9^-THC-induced hypoactivity (Fig. 5E), while a selective PPARγ antagonist (GW9662) produced a trend toward an increase in Δ^9^-THC-induced reduction in locomotion. A two-way RM ANOVA revealed a significant treatment X time interaction (Fig. 5E, F_22,308_ = 4.63, *p* < 0.001; Fig. 5F, F_22,308_ = 2.27, *p* < 0.001). The full statistical analysis results are shown in the supplementary Table 2. These findings suggest that PPAR mechanisms may not underlie cannabinoid action in locomotion.

### Effects of PPARα/γ antagonists on Δ^9^-THC-induced tetrad behavior

**Fig. 6:**
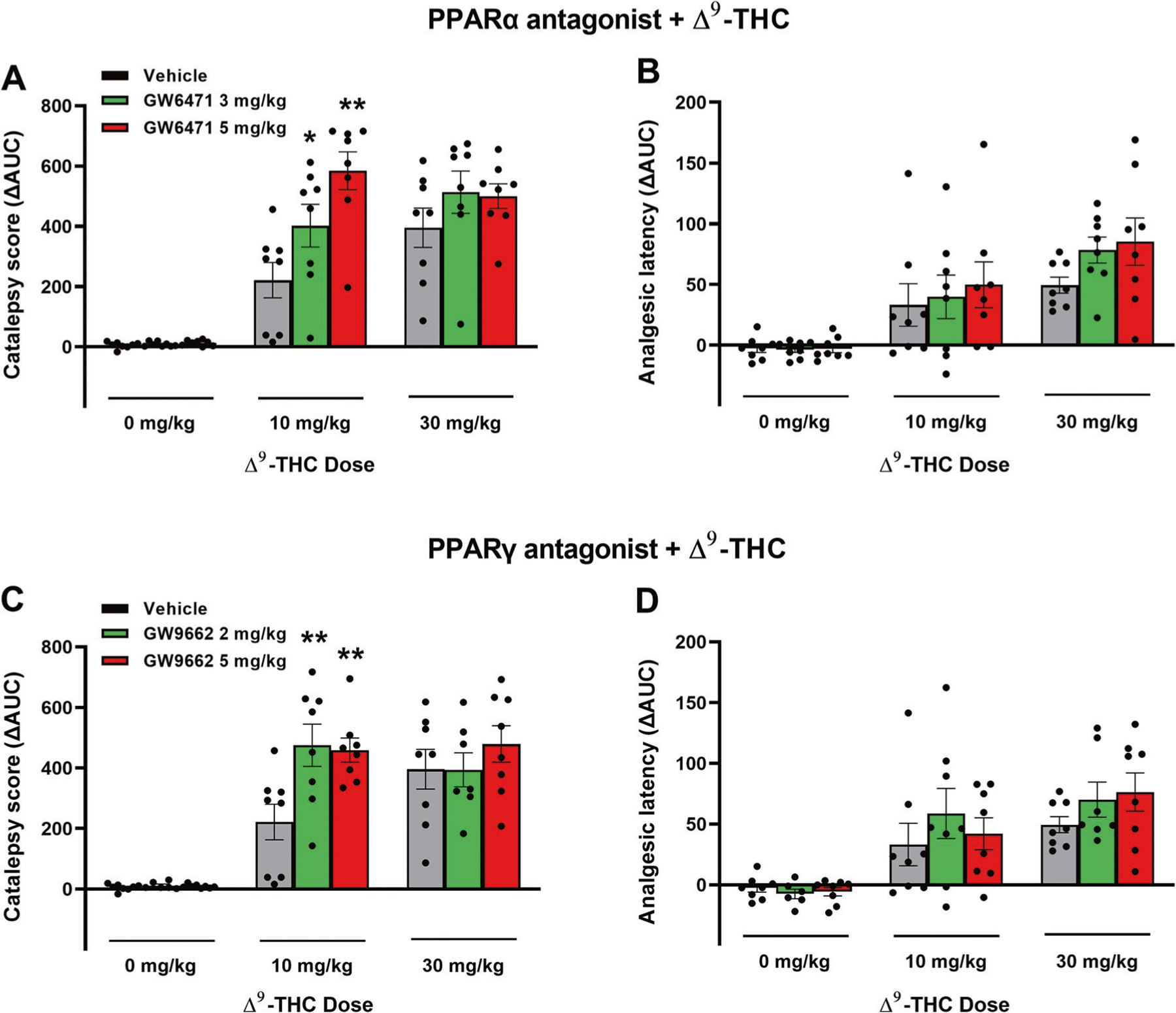
Effects of PPAR antagonists on Δ^9^-THC-induced catalepsy and analgesia in mice. **A/B** Pretreatment with the PPARα antagonist GW6471 enhanced 10 mg/kg Δ^9^-THC-induced catalepsy (**A**) but did not significantly alter hot-plate analgesia (**B**). **C/D** Pretreatment with the PPARγ antagonist GW9662 enhanced THC-induced catalepsy (**C**) but failed to alter Δ^9^-THC-induced analgesia (**D**). (See Figs. S8 and S9 for the effects of PPAR antagonists on Δ^9^-THC-induced hypothermia and immobility).

Lastly, we examined whether PPARs contribute to the classical tetrad effects of cannabinoids. Δ^9^-THC, at 10 and 30 mg/kg, produced prototypical cannabimimetic effects, e.g., catalepsy, analgesia, hypothermia, and rotarod locomotor impairment (i.e., immobility). The full time-course data are presented in Figs. S8 and S9. To make the data easier to view and understand, we provide graphs utilizing the changes in area under curve (ΔAUC) values (Fig. 6). We found that pretreatment with the selective PPARα antagonist GW6471 produced dose-dependent enhancement in Δ^9^-THC-induced catalepsy (Fig. 6A), a trend toward an increase in Δ^9^-THC-induced analgesia (Fig. 6B), but no effect on Δ^9^-THC-induced hypothermia or immobility (Figs. S8, S9). A two-way RM ANOVA on the catalepsy ΔAUC data revealed a significant main effect of Δ^9^-THC dose (Fig. 6A, F_2,21_ = 103.3, *p* < 0.001), GW6471 dose (F_2,21_ = 4.65, *p* < 0.05), and an interaction between these terms (F_4,42_ = 4.96, *p* < 0.05). Pairwise comparisons showed that Δ^9^-THC-induced catalepsy at 10 mg/kg was significantly enhanced by GW6471 (Fig. 6A). Similar two-way RM ANOVA’s were run for analgesia showing a significant main effect of Δ^9^-THC dose (*F*_2,21_ = 23.06; *P* < 0.001), but not of GW6471 dose (*F*_2,21_ = 1.51; *P* = 0.244) or the Δ^9^-THC x GW6471 interaction (*F*_4,42_ = 0.55; *P* = 0.703). Additional two-way RM ANOVA results for the full-time course data (Fig. S8) are provided in the supplementary Table 3.

Similarly, pretreatment with a PPARγ antagonist (GW9662) enhanced the cataleptic effects of Δ^9^-THC but had no effect on Δ^9^-THC-induced analgesia, hypothermia and immobility (Fig. 6C, D; Fig. S9). A two-way RM ANOVA on catalepsy scores revealed a significant Δ^9^-THC treatment main effect (Fig. 6C, F_2,21_ = 72.56, *p* < 0.001) and a significant Δ^9^-THC X GW9662 interaction (F_4,42_ = 3.05, *p* < 0.05), although no GW9662 main effect (F_2,21_ = 3.15, *p* = 0.064). Post-hoc comparisons detected a significant increase in 10 mg/kg Δ^9^-THC-induced catalepsy at both doses of GW9662 tested (2 and 5 mg/kg). Two-way RM ANOVAs on analgesic latency revealed significant main effects of Δ^9^-THC dose (*F*_2,21_ = 20.54; *P* < 0.001), but not of GW9662 dose (*F*_2, 21_ = 0.78; *P* = 0.455) or GW9662 X Δ^9^-THC interaction (*F*_4,42_ = 0.53; *P* = 0.716). Additional two-way RM ANOVA results for the full-time course data (Fig. S9) are provided in the supplementary Table 4.

## DISCUSSION

The major findings in this report include: (1) PPARα and PPARγ are mainly expressed on midbrain DA neurons, GABA and glutamate neurons in the amygdala, as well as on astrocyte-like cells in the NAc. (2) Optogenetic stimulation of VTA DA neurons is rewarding, which is dose-dependently inhibited by Δ^9^-THC or a PPARγ, but not PPARα, agonist, suggesting an important role of PPARγ in DA-dependent behavior. (3) PPARα and PPARγ antagonism weakened the reward-attenuating (aversive) and anxiogenic effects of Δ^9^-THC, potentiated Δ^9^-THC-induced hypoactivity and cataleptic properties, but failed to alter Δ^9^-THC-induced analgesia, hypothermia and immobility. These findings implicate PPARα and PPARγ in the VTA and amygdala in the affective profile of cannabinoids and DA-dependent behavior.

### PPARα and PPARγ expression in dopamine, glutamate and GABA neurons

Previous studies have investigated PPAR isotype mRNA and protein distribution in the rat brain [14, 43–45]. Double IHC assays have localized PPARα to neurons, astrocytes, and microglia and PPARγ to neurons and astrocytes in both human and mouse brains and in cultured rat neurons [29, 43, 46]. However, few studies have considered the phenotypes of neurons or cells that express PPARα and PPARγ in the mesolimbic reward system and amygdala. Early studies indicated PPARγ immunostaining in some DA neurons in the VTA [43], but not in GABA neurons in the rostromedial tegmental nucleus (RMTg) [14]. In the present report, we detected low densities of PPARα and PPARγ transcripts in DA, glutamate and GABA neurons in the VTA but high densities of PPARα or PPARγ immunostaining in ~70% of DA neurons, 30–40% of GABA neurons, and 10–20% of glutamate neurons in the VTA, suggesting an important role of PPARα and PPARγ in modulating DA-dependent behavior. As systemic administration of pioglitazone inhibited DA-dependent brain-stimulation reward (oICSS) in DAT-cre mice, the present findings suggest that dopaminergic PPARγ mechanisms may in part underlie the anti-reward effects of pioglitazone in experimental animals [14, 15].

Surprisingly, we detected PPARα and PPARγ in accumbal astrocyte-like cells, but not on GABAergic medium-spiny neurons. This finding is inconsistent with previous reports in which PPARα/γ-immunostaining was co-localized with primarily neuronal markers (NeuN or β-tubulin III), but not GFAP or Iba1 in the NAc and cortex [29, 46]. Further work is needed to address these conflicting findings.

It was previously reported that PPARγ transcripts are detected in GABA neurons in the hippocampus and amygdala [47]. Cannabinoids have biphasic anxiolytic and anxiogenic effects [5, 36], which are likely mediated by GABAergic and glutamatergic mechanisms in the amygdala, respectively [48, 49]. This inspired us to map out PPARα and PPARγ expression in the amygdala and determine their preferred neuronal subtypes. IHC assays indicated that PPARα was primarily expressed on glutamate neurons (57.3%) and PPARγ on GABA neurons (56.8%). These results are compatible with prior work and point to PPARs on both GABAergic and glutamatergic neurons in the amygdala as potential receptor mechanisms underlying the affective properties of cannabinoids.

We note that PPARα/γ transcription levels by RNAcope ISH assays were fairly low in all three brain regions assessed and an unusual pattern of expression was observed such that individual puncta were distributed within and outside of DAPI-labeled nuclei. In previous reports, similarly low transcription levels and expression patterns have been noted in the amygdala and hippocampus [16, 47]. It is not clear why mRNA levels are deficient relative to PPARα/γ-immunostaining. Further study is required to address this issue.

### PPARα/γ activation contributes to Δ^9^-THC-induced aversion

We have previously reported that cannabinoids produce a reduction in NAc DA release and DA-dependent oICSS in transgenic DAT-cre or VgluT2-cre mice [32, 36, 41, 42]. However, the receptor mechanisms underlying cannabinoid action in oICSS have not been explored in the above studies. In the present study, we found that pretreatment with a CB1 (AM251) or CB2 (AM630) receptor antagonist significantly blocked or reduced Δ^9^-THC-induced reduction in oICSS, suggesting that both membrane CB1 and CB2 receptors are critically involved in cannabinoid aversion. In addition to identification of CB1 and CB2 receptor expression in midbrain DA neurons [35, 36], we also identified PPARα and PPARγ in VTA DA neurons as discussed above. Furthermore, systemic administration of Δ^9^-THC or pioglitazone (a selective PPARγ agonist) dose-dependently inhibited oICSS, while pretreatment with a PPARγ antagonist significantly weakened the suppressive effect of Δ^9^-THC in this assay. These findings suggest that PPARγ activation also in part underlies Δ^9^-THC-induced reductions in oICSS. One possibility is that activation of PPARγ in midbrain DA neurons inhibits DA neuron activity and therefore DA-dependent oICSS. Another possibility is that PPARγ expressed in other types of neurons may also indirectly underlie Δ^9^-THC’s action in oICSS via unidentified neural circuits. We note that pioglitazone appears to be more potent than Δ^9^-THC in attenuation of oICSS. This is not the case as Δ^9^-THC, at a higher dose, produced more robust reduction in oICSS but also a significant reduction in open-field locomotion [50]. The latter finding suggests possible sedation or locomotor impairment, which complicates the data interpretation. Therefore, we didn’t include higher dose of Δ^9^-THC data in this study.

With PPARα, pharmacological activation failed to alter oICSS; however, pretreatment with a PPARα antagonist also reduced the suppressive effect of Δ^9^-THC on oICSS, suggesting that PPARα may modulate Δ^9^-THC aversion by transcript-mediated cellular changes in DA neurons and/or indirectly via unidentified neural circuits. Together, the above findings suggest that multiple receptor mechanisms, including membrane CB1 and CB2 and nuclear PPARs, underlie cannabinoid- or Δ^9^-THC-induced negative affection or aversion (Fig. 3C).

We note that blockade of PPARα/γ failed to alter Δ^9^-THC-induced CPA. There are several possible explanations. First, Δ^9^-THC is not a selective PPARγ agonist. It also binds to CB1, CB2 and GPR55 receptors [5, 7]. Thus, it is likely that blockade of a single receptor may not be sufficient to block Δ^9^-THC-induced place aversion. Second, the CPP/CPA test does not directly measure the acute rewarding or aversive effects of cannabinoids. Instead, it assesses reward-associated learning and memory captured at least 24 h after the last Δ^9^-THC administration. As such, different neural mechanisms may underlie Δ^9^-THC-induced reduction in oICSS versus place aversion. Third, CPP/CPA experiments are infamously insensitive to subtle changes in drug reward [51, 52]. In contrast, oICSS is highly sensitive to small changes in brain reward function [32]. Lastly, oICSS provides a microcosm of a drug effect on a specific phenotype of neurons in a specific brain area, while place conditioning conveys the larger picture: the generally negative or positive associations an animal develops after repeated experiences to a drug. To summarize, both the oICSS and CPP assays are examining quantitatively and qualitatively distinct endpoints and a negative finding in a CPP test may not necessarily conflict with the positive finding in oICSS. Interestingly, GW9662 (a PPARγ antagonist), at 2 mg/kg, produced a significant CPA (Fig. S6E). However, given that GW9662 failed to alter DA-mediated oICSS (Fig. 2L), it is suggested that an indirect non-DA mechanism may be involved.

In prior work, both PPARγ and PPARα agonists were reported to decrease the reinforcing value and motor-stimulating effects of drugs of abuse including nicotine, ethanol, heroin, and methamphetamine [14, 15, 53, 54]. However, the neural mechanisms underlying this action are poorly understood. Previous studies indicate that the PPARα agonists (WY14643 and methOEA) and the PPARγ agonist (pioglitazone) prevented nicotine- and morphine-induced increases in DA neuron firing in the VTA [14, 53]. A presynaptic GABAergic PPARγ mechanism has been proposed to explain the above finding in DA [14]. However, this hypothesis is not supported by their finding that PPARγ is not expressed in RMTg GABA neurons [14]. Little is known whether PPARγ modulates GABA neuron activity or GABA release in the RMTg or VTA. In the present study, we found that both PPARα and PPARγ are expressed in most of VTA DA neurons (Fig. 1) and PPARγ agonism inhibits DA-mediated oICSS (Fig. 3C). It is suggested that dopaminergic PPAR mechanisms at least in part explain how PPAR agonists produce therapeutic effects against drug reward. As PPARα and PPARγ are also expressed in both GABA neurons and glutamate neurons in the VTA and amygdala, PPAR mechanisms in other brain region non-DA neurons may also contribute to the pharmacological action produced by PPAR agonists in animal addiction models.

### PPARs contribute to Δ^9^-THC-induced anxiety

Another important finding in this report is that antagonism of PPARα and PPARγ attenuated Δ^9^-THC-induced anxiety, supporting the above finding that both receptors are indeed functionally involved in the negative affective properties of cannabinoids. This is consistent with previous work indicating that PPARγ is critically involved in stress sensitivity and anxiety [47, 55, 56]. For example, PPARγ-KO mice developed enhanced emotional response to stress and exacerbated anxiety [47]. PPARγ agonism was reported to attenuate nicotine withdrawal-induced anxiety and somatic signs [16], suggesting that PPARγ agonists may have therapeutic potential against substance use disorders.

We note that PPARα/γ agonists or antagonists alone failed to alter basal anxiety levels, while PPARα or PPARγ antagonism only partially reduced Δ^9^-THC-induced anxiety. These findings mirror earlier assessments in which activation of PPARs only modulated anxiety in response to lipopolysaccharide exposure or restraint stress but did not alter basal anxiety levels [47, 55, 56].

### PPARs modulate Δ^9^-THC-induced hypoactivity and catalepsy

A third important finding is that both PPARα and PPARγ modulate basal level locomotion: the PPARγ agonist produced a transient increase, while both the PPAR antagonists produced a robust decrease in open-field locomotion. In agreement with these findings, pretreatment with a PPARα antagonist, but not with a PPARγ antagonist, potentiated Δ^9^-THC-induced hypoactivity, suggesting that PPARα antagonism produced an additive or synergistic effect with Δ^9^-THC in open-field locomotion. In addition, pretreatment with PPARα or PPARγ antagonists also potentiated Δ^9^-THC-induced catalepsy. Neither PPARα nor PPARγ antagonists altered Δ^9^-THC-induced analgesia, hypothermia, or immobility. These findings suggest that PPARα and PPARγ are functionally involved in a subset of cannabinoid CNS effects. The precise mechanisms through which PPARs modulate motor function remain to be determined.

In conclusion, in this study we systemically evaluated the cellular expression of PPARα and PPARγ in the brain and their functional roles in the CNS effects of Δ^9^-THC. We found that PPARα and PPARγ are expressed in midbrain DA neurons and in both GABA and glutamate neurons in the amygdala. Activation of PPARγ inhibits DA-dependent oICSS, while blockade of PPARα and PPARγ attenuates Δ^9^-THC-induced reward-attenuation and anxiety but potentiates Δ^9^-THC-induced hypoactivity and catalepsy. These results provide novel insights regarding the role of PPARα and PPARγ in cannabis action and highlight the potential utility of PPARs as new therapeutic targets for substance use disorders.

## Supplementary Material

Fig. S1

Fig. S3

Fig. S2

Fig. S5

Fig. S6

Fig. S4

Fig. S7

Fig. S8

Fig. S9

Suppl Table 1

Suppl Table 3

Suppl Table 2

Suppl Table 4

Suppl. Materials

## Data Availability

The raw data in this paper is available upon request.
