# Supplementary figures and images for: PPARα and PPARγ are expressed in midbrain dopamine neurons and modulate dopamine- and cannabinoid-mediated behavior in mice
Source: Mol Psychiatry. Author manuscript; Available in PMC 2024 Apr 1. (PMC10799974; doi:10.1038/s41380-023-02182-0)

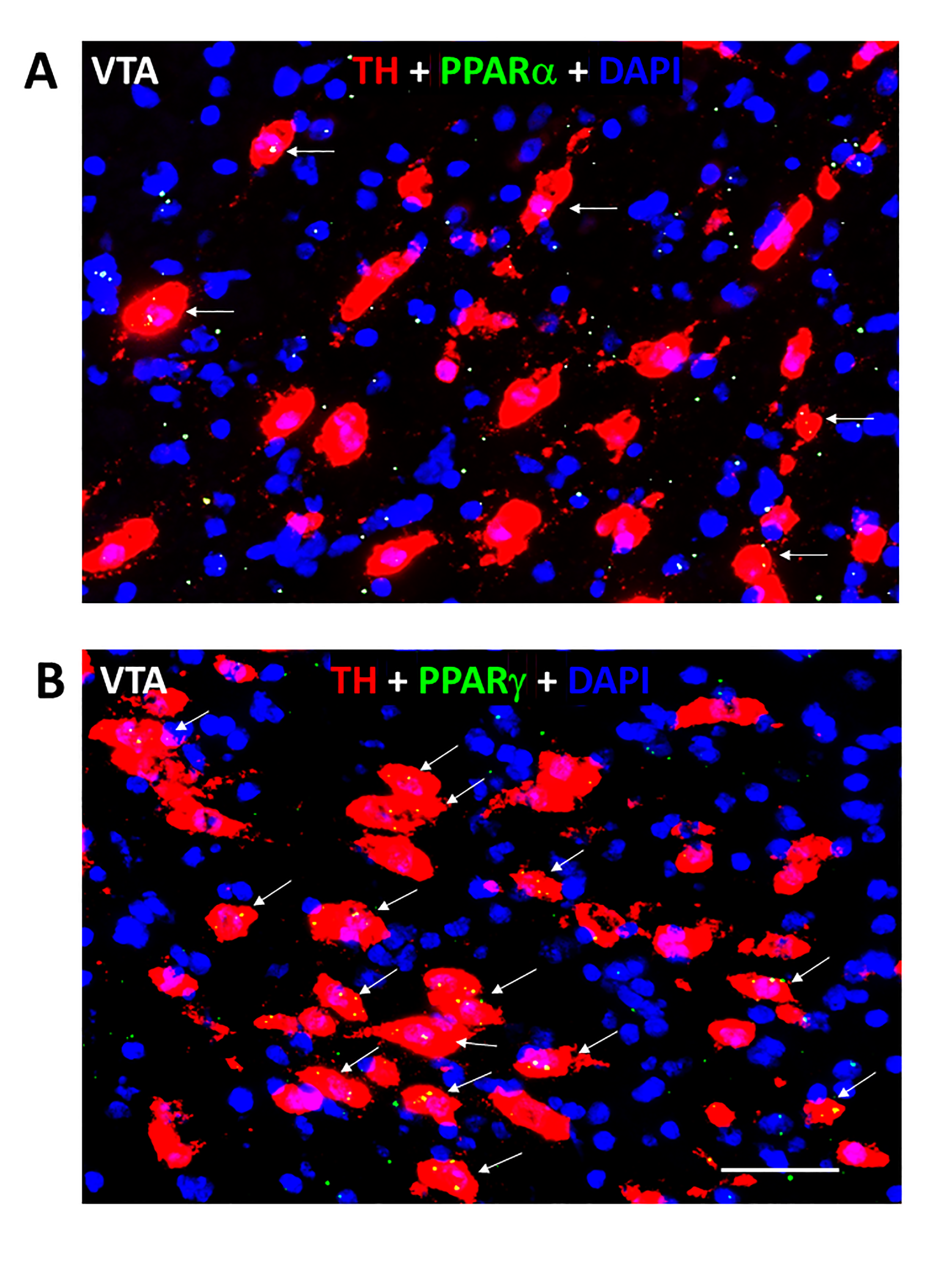

Supplement: Fig. S1 [file NIHMS1947749-supplement-Fig__S1.tif]

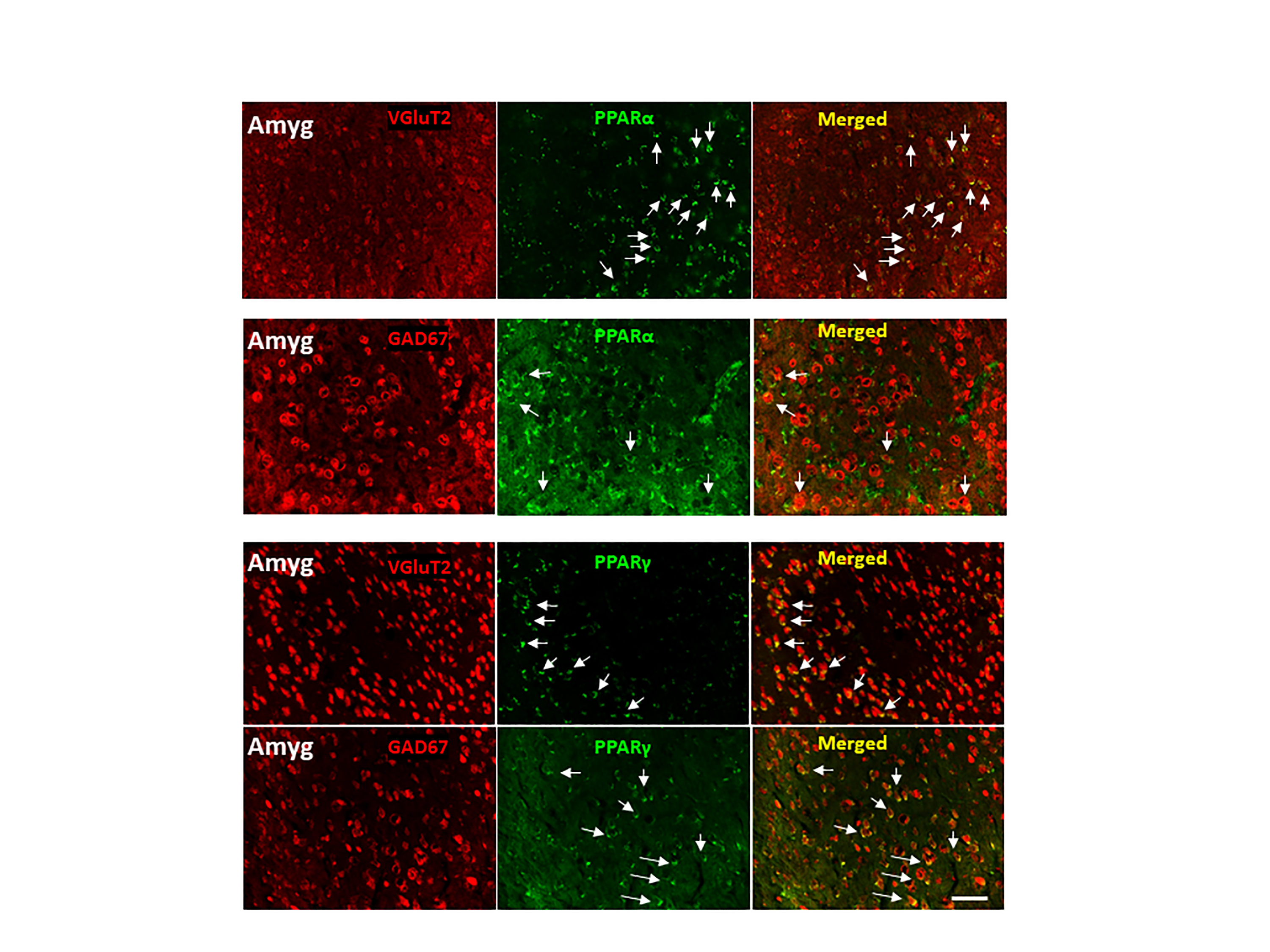

Supplement: Fig. S5 [file NIHMS1947749-supplement-Fig__S5.tif]

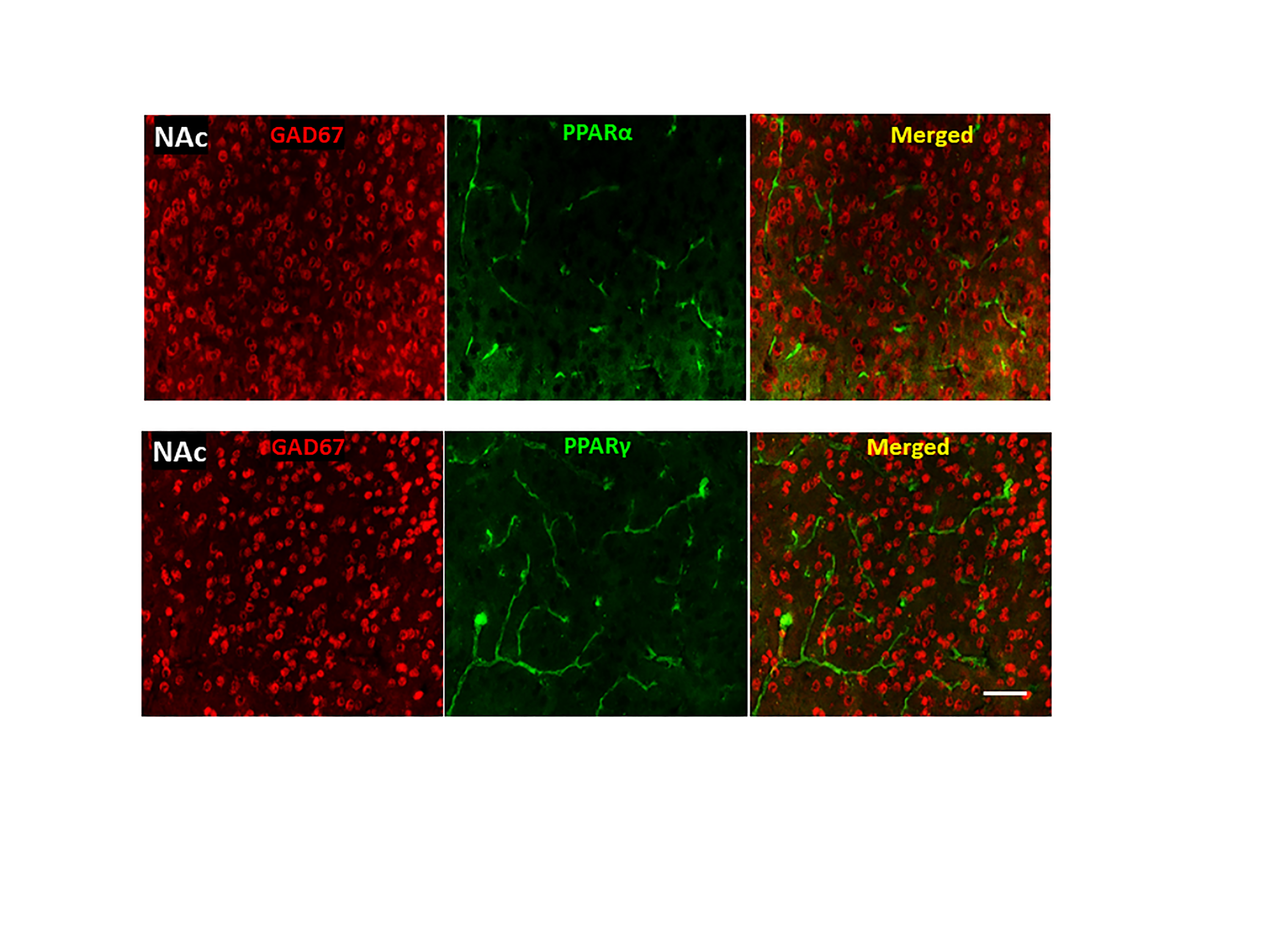

Supplement: Fig. S6 [file NIHMS1947749-supplement-Fig__S6.tif]

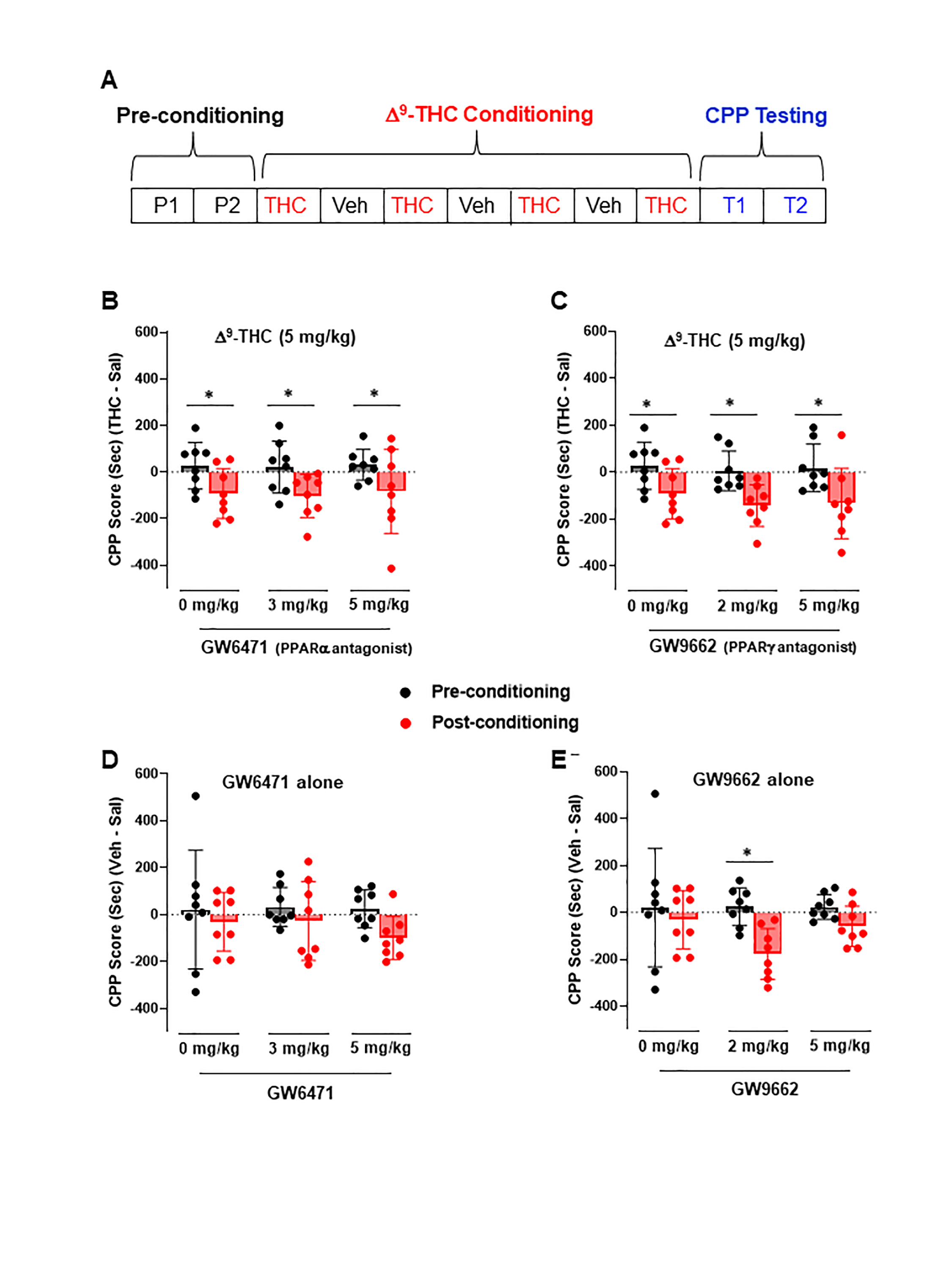

Supplement: Fig. S7 [file NIHMS1947749-supplement-Fig__S7.tif]

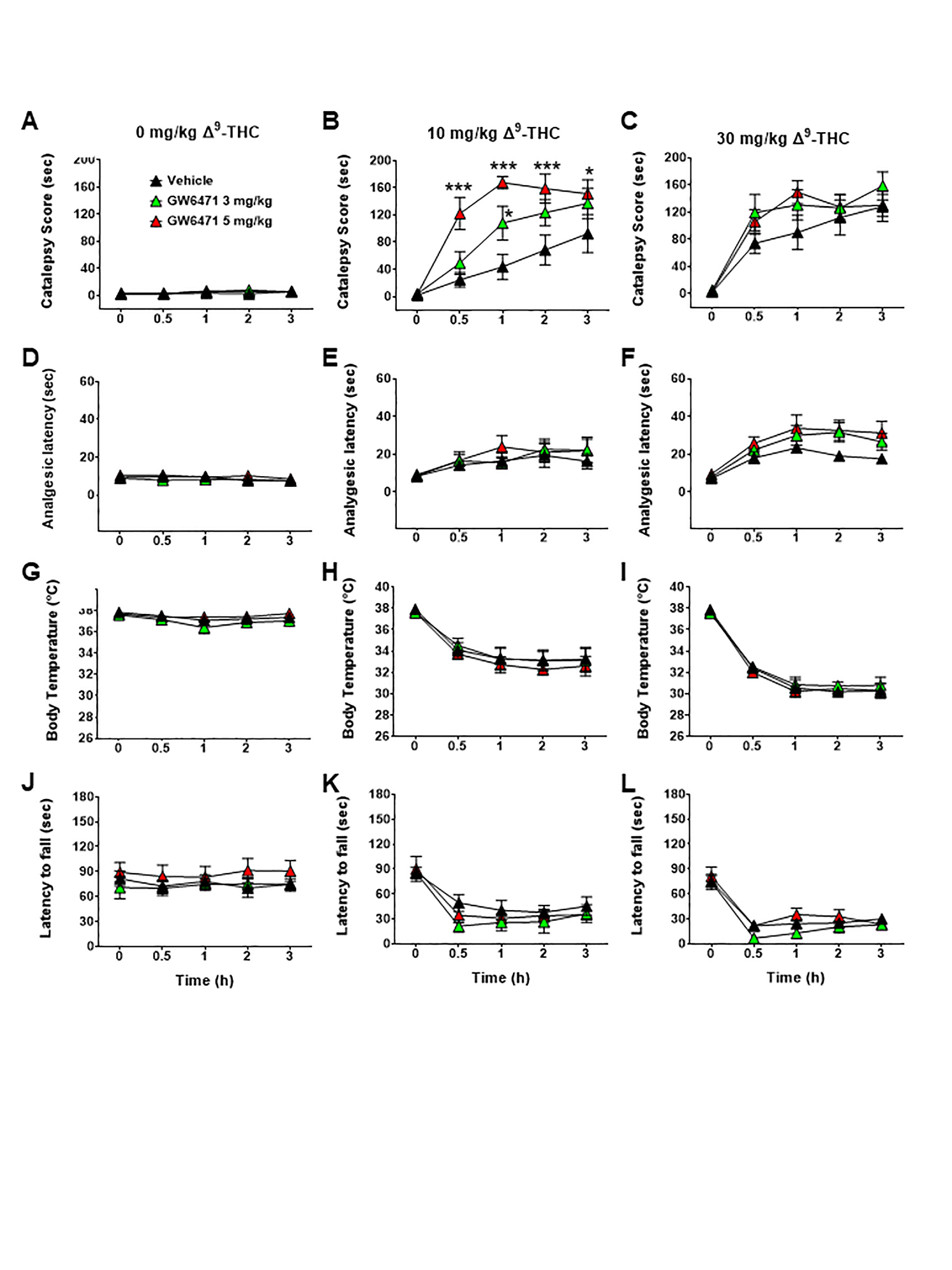

Supplement: Fig. S8 [file NIHMS1947749-supplement-Fig__S8.tif]

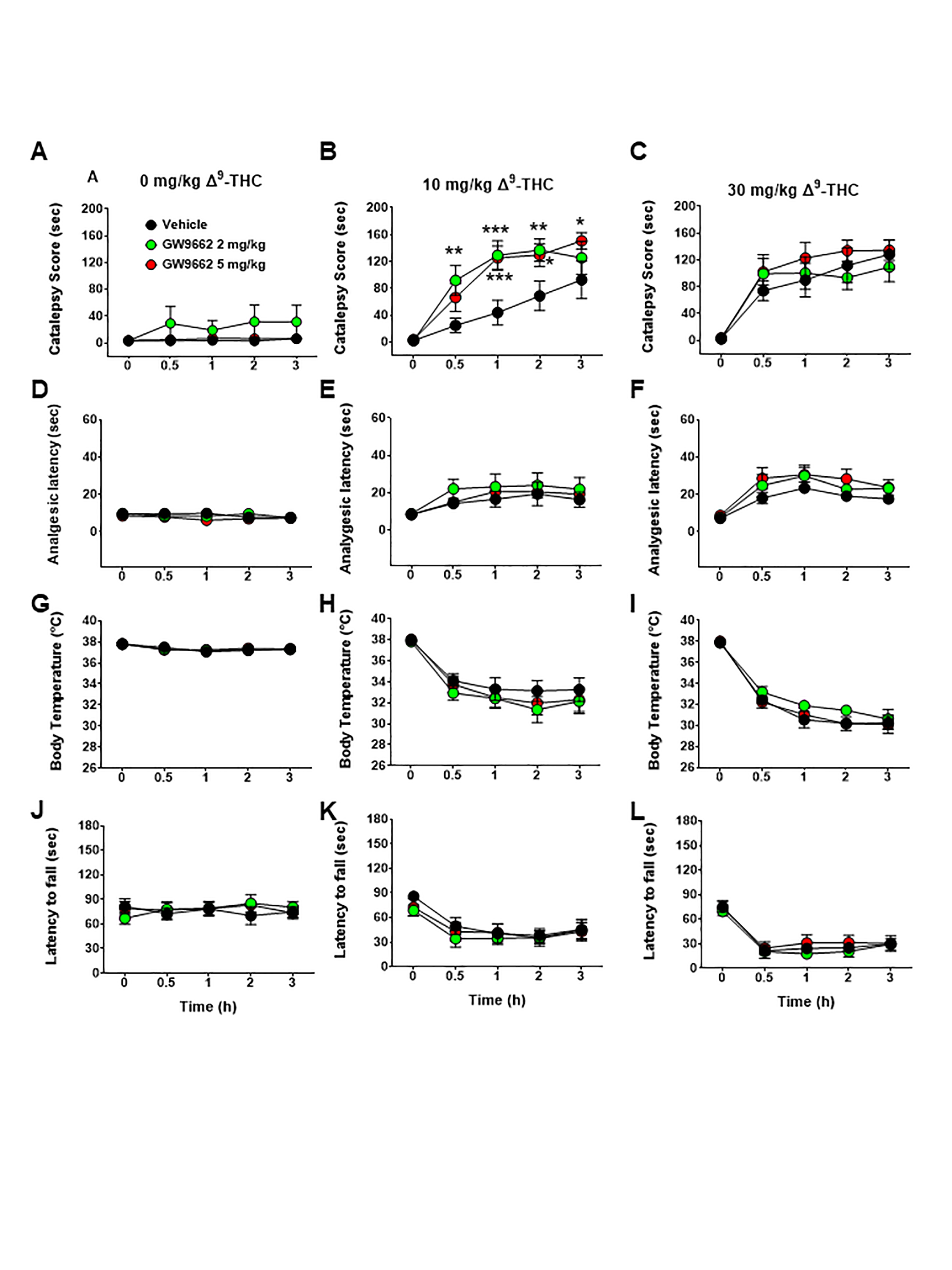

Supplement: Fig. S9 [file NIHMS1947749-supplement-Fig__S9.tif]
