# Supplementary material for: PPARα and PPARγ are expressed in midbrain dopamine neurons and modulate dopamine- and cannabinoid-mediated behavior in mice
Source: Mol Psychiatry. Author manuscript; Available in PMC 2024 Apr 1. (PMC10799974; doi:10.1038/s41380-023-02182-0)
Supplement: Suppl Table 1 [file NIHMS1947749-supplement-Suppl_Table_1.docx]

**Supplementary Table 1.**Statistical analysis results by RM ANOVAs over time and drug dose

| **Figure**[**2**](https://bpspubs.onlinelibrary.wiley.com/doi/10.1111/bph.14958#bph14958-fig-0001)  (oICSS) | **Drug treatment**  **main effect** | **Time (Hz) main effect** | **Treatment × time**  **interaction** |
| --- | --- | --- | --- |
| Fig. 2D | *F*_2, 49_ = 5.185; *P* <0.01 | *F*_5, 245_= 117.2; *P* <0.001 | *F*_10, 245_ = 2.53; *P* <0.01 |
| Fig. 2E | *F*_3, 37_ = 0.4403; *P* =0.7255 | *F*_5, 185_ = 123.1; *P* <0.001 | *F*_15, 185_ = 0.43; *P* =0.969 |
| Fig. 2F | *F*_3,41_ = 8.15; *P* <0.001 | *F*_5, 205_ = 78.49; *P* <0.001 | *F*_15, 205_ = 7.59; *P* <0.001 |
| Fig. 2G | *F*_3, 60_ = 3.83; *P* <0.05 | *F*_5, 300_ = 161.9; *P* <0.001 | *F*_15, 300_ = 2.64; *P* <0.001 |
| Fig. 2I | *F*_2, 33_ = 0.044; *P* =0.957 | *F*_5, 165_ = 153.9; *P* <0.001 | *F*_10, 165_ = 0.23; *P* =0.993 |
| Fig. 2J | *F*_3, 60_ = 3.795; *P* <0.05 | *F*_5, 300_ = 168.4; *P* <0.001 | *F*_15, 300_ = 2.41; *P* <0.01 |
| Fig. 2L | *F*_2, 33_ = 4.582; *P* <0.05 | *F*_5, 165_ = 108.4; *P* <0.001 | *F*_10, 165_ = 4.55; *P* <0.001 |
