# Supplementary material for: PPARα and PPARγ are expressed in midbrain dopamine neurons and modulate dopamine- and cannabinoid-mediated behavior in mice
Source: Mol Psychiatry. Author manuscript; Available in PMC 2024 Apr 1. (PMC10799974; doi:10.1038/s41380-023-02182-0)
Supplement: Suppl Table 2 [file NIHMS1947749-supplement-Suppl_Table_2.docx]

**Supplementary Table 2.**Statistical analysis results by two-way RM ANOVAs over time and drug dose

| **Figure**[**5**](https://bpspubs.onlinelibrary.wiley.com/doi/10.1111/bph.14958#bph14958-fig-0001)  (Locomotion) | **Drug treatment**  **main effect** | **Time main effect** | **Treatment × time**  **interaction** |
| --- | --- | --- | --- |
| Fig. 5A | *F*_2,21_ = 1.28; *P* >0.05 | *F*_11,231_ = 5.78; *P* <0.001 | *F*_22,231_ = 3.48; *P* <0.001 |
| Fig. 5B | *F*_2,21_ = 0.44; *P* >0.05 | *F*_11,231_ = 13.05; *P* <0.001 | *F*_22,231_ = 5.36; *P* <0.001 |
| Fig. 5C | *F*_2,21_ = 17.39; *P* <0.001 | *F*_11,231_ = 7.84; *P* <0.001 | *F*_22,231_ = 4.78; *P* <0.001 |
| Fig. 5D | *F*_2,21_ = 5.67; *P* <0.01 | *F*_11,231_ = 8.15; *P* <0.001 | *F*_22,231_ = 3.49; *P* <0.001 |
| Fig. 5E | *F*_3,28_ = 5.86; *P* < 0.01 | *F*_11,308_ = 15.37; *P* <0.001 | *F*_33,308_ = 4.65; *P* <0.001 |
| Fig. 5F | *F*_3,28_ = 3.67; *P* < 0.05 | *F*_11,308_ = 10.02; *P* <0.001 | *F*_33,308_ = 2.27; *P* <0.001 |
