# Supplementary material for: PPARα and PPARγ are expressed in midbrain dopamine neurons and modulate dopamine- and cannabinoid-mediated behavior in mice
Source: Mol Psychiatry. Author manuscript; Available in PMC 2024 Apr 1. (PMC10799974; doi:10.1038/s41380-023-02182-0)
Supplement: Suppl Table 4 [file NIHMS1947749-supplement-Suppl_Table_4.docx]

**Supplementary Table 4.**Statistical analysis results by two-way ANOVAs for repeated measures over time and GW9662 dose

| **Figure S9**  (Δ^9^-THC tetrad) | **Drug treatment**  **main effect** | **Time main effect** | **Treatment × time**  **interaction** |
| --- | --- | --- | --- |
| Fig. S9-A | *F*_2, 21_ = 1.36; *P* =0.278 | *F*_4, 84_ = 2.11; *P* =0.087 | *F*_8, 84_ = 1.52; *P* =0.163 |
| Fig. S9-B | *F*_2, 21_ = 6.00; *P* <0.01 | *F*_4, 84_ = 32.21; *P* <0.001 | *F*_8, 84_ = 1.80; *P* =0.088 |
| Fig. S9-C | *F*_2, 21_ = 0.76; *P* =0.480 | *F*_4, 84_ = 29.04; *P* <0.001 | *F*_8, 84_ = 0.56; *P* =0.811 |
| Fig. S9-D | *F*_2, 21_ = 1.32; *P* =0.290 | *F*_4, 84_ = 2.73; *P* <0.05 | *F*_8, 84_ = 1.57; *P* =0.148 |
| Fig. S9-E | *F*_2, 21_ = 0.52; *P* =0.601 | *F*_4, 84_ = 8.68; *P* <0.001 | *F*_8, 84_ = 0.31; *P* =0.961 |
| Fig. S9-F | *F*_2, 21_ = 2.17; *P* =0.139 | *F*_4, 84_ = 23.49; *P* <0.001 | *F*_8, 84_ = 0.53; *P* =0.830 |
| Fig. S9-G | *F*_2, 21_ = 0.07; *P* =0.934 | *F*_4, 84_ = 13.11; *P* <0.001 | *F*_8, 84_ = 0.67; *P* =0.714 |
| Fig. S9-H | *F*_2, 21_ = 0.46; *P* =0.638 | *F*_4, 84_ = 57.65; *P* <0.001 | *F*_8, 84_ = 0.41; *P* =0.909 |
| Fig. S9-I | *F*_2, 21_ = 1.01; *P* =0.383 | *F*_4, 84_ = 184.38; *P* <0.001 | *F*_8, 84_ = 0.75; *P* =0.649 |
| Fig. S9-J | *F*_2, 21_ = 0.053; *P* =0.948 | *F*_4, 84_ = 0.37; *P* =0.832 | *F*_8, 84_ = 1.37; *P* =0.223 |
| Fig. S9-K | *F*_2, 21_ = 0.26; *P* =0.775 | *F*_4, 84_ = 20.92; *P* <0.001 | *F*_8, 84_ = 0.49; *P* =0.863 |
| Fig. S9-L | *F*_2, 21_ = 0.29; *P* =0.744 | *F*_4, 84_ = 90.69; *P* <0.001 | *F*_8, 84_ = 0.54; *P* =0.825 |
