# Supplementary material for: PPARα and PPARγ are expressed in midbrain dopamine neurons and modulate dopamine- and cannabinoid-mediated behavior in mice
Source: Mol Psychiatry. Author manuscript; Available in PMC 2024 Apr 1. (PMC10799974; doi:10.1038/s41380-023-02182-0)
Supplement: Suppl. Materials [file NIHMS1947749-supplement-Suppl__Materials.pdf]

Molecular Psychiatry

Supplementary Information

PPARα and PPARγ are expressed in midbrain dopamine neurons and modulate dopamine- and cannabinoid-mediated behavior in mice

Briana Hempel[1,2], Madeline Crissman[3], Sruti Pari[3], Benjamin Klein[1,2], Guo-Hua Bi[1,2], Hannah Alton[1,2], Zheng-Xiong Xi [1]

Corresponding Author: Zheng-Xiong Xi, 251 Bayview Boulevard, Baltimore, MD 21224. Phone: (443) 740-2517. Email: zxi@intra.nida.nih.gov

MATERIALS AND METHODS

Experiment 1: RNAscope in situ hybridization

Mice were sacrificed via rapid decapitation and brains were wrapped in aluminum foil on dry ice and stored in a -80°C freezer until use. Coronal sections were taken at 16 μm thickness and mounted onto positively charged glass slides (Fisherbrand Superfrost Plus; Fisher Scientific, Pittsburgh, PA). Slides were incubated in 10% cold formalin for 20 min, washed in PBS (x2), and dehydrated in graduated ethanol (50%, 75% and 100%). Samples were prepared using the manufacturer's instructions for the RNAscope Multiplex Fluorescent Reagent Kit v2 (Advanced Cell Diagnostics, Newark, CA). During pretreatment, slides were incubated in hydrogen peroxide (10 min, room temperature), washed in dH_2O (x2), incubated in Protease-Pretreat IV solution (20 min, room temperature), and washed in PBS (x2). The appropriate mRNA probes were then added to the slides, hybridized in the HybEZ oven (40°C) for 2 hrs and washed with a prepared RNAscope wash buffer (x2). Three additional hybridization steps in the oven followed with AMP1 (30 min), AMP2 (30 min), and AMP3 (15 min) solutions applied to the slides and rinsed with wash buffer (x2) between each step. Next, each individual probe signal was developed using Opal Dyes (Akoya Biosciences, Marlborough, MA). For a given channel (1, 2 and/or 3), HRP-C(n) was added to the slides (15 min, 40°C), followed by a diluted Opal dye (520, 570 or 690; 30 min, 40°C), and HRP blocker (15 min, 40°C) with 2 buffer washes between each addition. The following dilution ratios were utilized for each probe: 1:750 (*PPARA:* Cat No. 454051*, PPARG:* 418821), 1:1500 (*GAD1*:511931-C2, 511931-C3*, Slc17a6: 428871-C2*), and 1:3000 (*TH: 317621-C3*). After a short incubation with DAPI (30 s), each slide received fluorescent mounting medium (Fluoro-

Gel; #17 985, Electron Microscopy Science) and a coverslip. A Keyence BZ-X800 Fluorescence Microscope was used to take images at 60x magnification.

Experiment 2: Immunofluorescence

Mice were anesthetized with isoflurane gas and intracardially perfused with ice-cold 0.9% saline then 4% PFA. Brains were removed and placed in 4% PFA overnight. The following morning, the tissue was transferred to a 30% sucrose solution. After 48 hr in sucrose, brains were frozen and sliced in 30 μm coronal sections. Free floating slices were rinsed in PB (x3, 10 min each) and blocked in a 5% donkey serum and 0.3% Triton X-100 solution in PB for 1 hr with agitation. Sections were incubated overnight at 4°C on a shaker with two primary antibodies in PB containing 3% donkey serum and 0.3% Triton X-100. The following antibodies were selected: rabbit anti-PPARα (1:200; ThermoFisher Scientific, PA1-822A), rabbit anti-PPARγ (1:250; Abcam, ab45036), sheep anti-TH (1:1500; ThermoFisher Scientific, PA1-4679), GAD67 anti-chicken (1:200; Abcam, ab75712), and VGluT2 anti-chicken (1:500; Synaptic Systems, 135 416). Sections were rinsed in PB (x3, 10 min each) and carefully transferred to a cocktail of two fluorescent labeled secondary antibodies including Alexa Fluor 488 Donkey Anti-Sheep (1:1500; Abcam, ab150177) Alexa Fluor 594 Donkey Anti-Rabbit (1:500; Abcam, ab150076), and Alexa Fluor 647 Donkey Anti-Chicken (1:500; Jackson ImmunoResearch, 703-605-155) for two hours at room temperature with agitation. After another round of rinses (x3, 10 min each), slices were incubated with DAPI for 5 minutes in PB (1 drop per 500 μL) and mounted on gelatin-coated slides. Fluorescent

mounting medium was applied, and slides were cover slipped and allowed to dry in a dark place.

Images were obtained using a Keyence BZ-X800 Fluorescence Microscope at 40x magnification. For cell counting analyses, sections were acquired from 3 mice and 6 brain slices were selected from each brain. 2-4 images were taken per brain section, depending on the size of the region. DAPI-positive cells that expressed PPARα or PPARγ in combination with TH, GAD67, or VGluT2 were counted using Keyence image analysis software.

Experiment 3: Optical Intracranial self-stimulation

***Surgery**:* Male and female DAT-cre mice were anesthetized with a ketamine (100 mg/kg) xylazine (10 mg/kg) mixture and placed in a stereotaxic device (David Kopf Instruments, Tujunga, CA). Subjects received bilateral microinjections of an excitatory channelrhodopsin virus (AAV5-EF1α-DIO-ChR2-EYFP, University of North Carolina Gene Therapy Center) in the VTA (AP -3.28, ML ± 0.43, DV -4.41; 10° angle from the midline). Infusions were delivered at a rate of 50 nl/min (150 nl per side) using two 10 μl stainless steel syringes (Nanofil) and a Micro 4 Pump (World Precision Instruments). Injectors remained in place for an additional 5 minutes following AAV delivery. Custom built ferrule fibers (200 μM diameter, Thorlabs, Newton, NJ) were implanted 0.5 mm above the virus injection sites and secured to the skull using dental cement. Four weeks separated the surgical procedures from the start of testing to facilitate viral expression.

***oICSS Procedure*:** Subjects were tested in operant conditioning chambers (Med Associates, Fairfax, VT) with two retractable levers, cue light panels, a house light, and a speaker. A patch cord (Doric Lenses Inc., Quebec, QC, Canada) on a swivel connected subject's ferrule head mounts to a 473 nm laser (OEM Laser Systems, Inc., Draper, UT). Initially, mice were placed on an FR1 schedule in which a single response on the active lever led to a 1-s pulse of laser stimulation (473 nm, 20 mW, 5 ms duration, 50 Hz) and a 1-s activation of the cue light above the active lever. Responses on the inactive lever had no programmed consequences. Daily sessions were 60 min in length. Once stable lever pressing was observed, a rate-frequency oICSS procedure was instituted. In this program, 6 stimulation frequencies (100, 50, 25, 10, 5, 1 Hz) were available in descending order during sessions, with a new frequency presented every 10 minutes.

Initially, we tested one group of mice with Δ^9-THC (1 & 3 mg/kg IP; 15 min prior to testing) and found that 3 mg/kg shifted the response-frequency curve to the right without suppressing locomotor activity. We then divided the animals into two groups to examine the effects of PPARα and PPARγ antagonists on 3 mg/kg Δ^9-THC-induced changes in oICSS, respectively. GW6471 (a selective PPARα antagonist, 3 & 5 mg/kg) and GW9662 (a selective PPARγ antagonist, 2 & 5 mg/kg) were delivered IP 15 minutes prior to 3 mg/kg Δ^9-THC and after a 15 min drug absorption period, mice were placed in the oICSS chambers for testing. PPAR antagonists or agonists alone were also tested individually. Specifically, different doses of GW6471 (3 & 5 mg/kg), GW9662 (2 & 5 mg/kg), GW7647 (a selective PPARα agonist, 3, 5, 10 mg/kg) and Pioglitazone (a selective PPARγ antagonist, 3, 5, 10 mg/kg) were administered 15 minutes prior to a

typical oICSS session. Tests were conducted 3-5 days apart. The order of testing with different doses of a drug was counterbalanced.

Experiment 4: Conditioned place preference or aversion (CPP/CPA)

Subjects underwent an 11-day place conditioning regimen. During the preconditioning (Day 1 & 2) and postconditioning (Day 10 & 11) phase, mice were placed in the center aisle of a place conditioning apparatus and allowed to explore all three compartments for 15 min. Time spent in each chamber was calculated. On days 3-9, subjects were restricted to one of the two large chambers for 45 min conditioning sessions. On drug days (3,5,7,9), different groups of mice were randomly pretreated with vehicle, GW6471 (3, 5 mg/kg) or GW9662 (2 or 5 mg/kg), respectively, 15 minutes prior to Δ^9-THC (0 or 5 mg/kg) injections and then immediately placed in one of the conditioning chambers. On saline days (4,6,8), subjects received two saline injections 15 minutes apart and entered the opposite chamber. The drug associated compartment was randomly selected.

Experiment 5: Elevated Plus Maze

Mice were acclimated to the testing room containing the maze for 1 hr and administered IP injections in a separate area. The testing room was dark with illumination provided by two standing lamps with red lightbulbs placed on opposite sides of the maze. After habituation, different groups of mice (9-13 per group) were injected with vehicle, GW6471 (3, 5 mg/kg), or GW9662 (2, 5 mg/kg) followed by a 15 min drug

absorption period. An anxiogenic dose of Δ^9-THC (5 mg/kg) or vehicle was administered, and 30 min post injection mice were placed in the center of the EPM for five minutes. EthoVision software recorded time spent in the open and closed arms of the maze. We were also interested in whether activation or inhibition of PPARα or PPARγ produces changes in anxiety in the absence of Δ^9-THC. To test this, the PPAR agonists pioglitazone and GW7647 (5 & 10 mg/kg) and the PPAR antagonists GW6471 (3 & 5 mg/kg) and GW9662 (2 & 5 mg/kg) were tested alone in the EPM. Mice were injected 15 min prior to placement in the maze. Tests were conducted 3-5 days apart. The order of testing with different doses of a drug was counterbalanced.

Experiment 6: Open field Locomotion

Two groups of WT mice (n=8/group) were used to evaluate the locomotor effects of PPARα and PPARγ compounds. Subjects were initially given a single 2-hr session to habituate to the chambers. On testing days, the PPARα antagonist GW6471 (0, 3, 5 mg/kg) or the PPARγ antagonist GW9662 (0, 2, 5 mg/kg) was administered 15 minutes prior to injections of vehicle (5% cremophor) or 3 mg/kg Δ^9-THC. Animals were placed in the open field chambers for 2 hrs immediately following Δ^9-THC injections. The dose of Δ^9-THC was selected based on a pilot study demonstrating a significant decrease in open field activity, without altering performance on the rotarod. We were also interested in whether manipulations of the PPAR by itself would affect locomotion. To assess this, four additional groups of mice (n = 8/group) were administered a PPARα agonist (5 and 10 mg/kg GW7647), PPARγ agonist (5 and 10 mg/kg Pioglitazone), PPARα antagonist (3 and 5 mg/kg GW6471), or a PPARγ antagonist and placed in the open-field

chambers for 2 hrs. Tests were conducted 3-5 days apart. The order of testing with different doses of a drug was counterbalanced.

Experiment 7: Δ^9-THC-induced Tetrad

Five groups of mice (n = 8 per group) were pretreated with vehicle, a PPARα (3 or 5 mg/kg GW6471), or PPARγ (2 or 5 mg/kg GW9662) antagonist and then were administered Δ^9-THC. Baseline (time 0) data was collected on a series of four behavioral tests commonly referred to as 'the tetrad' [1-3] to measure cannabinoid-induced analgesia, catalepsy, hypolocomotion, and hypothermia. The order of testing was randomized to prevent order effects. Each mouse received one of the five pretreatment injections followed by a 15 minute drug absorption period. Next, Δ^9-THC (0, 10, or 30 mg/kg) was administered, and tetrad assessments were taken 0.5, 1, 2 and 3 hrs. post injection. Subjects were assigned to a single pretreatment group but received all three doses of Δ^9-THC in a random order with three washout days between rounds of testing. ***Catalepsy**:* The elevated bar test evaluated cataleptic behavior. Subjects' front paws were arranged on a metal bar at a height to which their hind paws were just able to reach the ground. The latency for mice to reorient themselves and place both front paws on the ground was timed with a cut off period of 180 s.

***Analgesia**:* Analgesia was assessed using a hot plate device (Model 39, IITC Life Science Inc., CA). Mice were placed on a platform heated to 52°C with a transparent barrier in place and the latency to the first sign of pain was recorded. Thermal nociceptive signs included paw licking, stomping hind paws, or jumping.

Animals were removed from the platform immediately following these behaviors or when the 60s duration was reached to avoid tissue damage.

Hypothermia*:* A rectal thermometer measured decreases in body temperature (°C). The RET-2 rectal probe (Harvard Apparatus, Holliston, MA) was lubricated and gently inserted 2 cm into the rectum.

Immobility: Decreased coordination and mobility was assessed with a rotarod device (Harvard Apparatus). Subjects were trained for four days prior to the start of tetrad procedures. They were initially placed on the rotating bar at a low speed (5 revolutions per minute) which was incrementally increased until animals were able to maintain a 20 rev/min speed for at least 1 minute without falling. During testing, the rotarod program gradually increased speed from 4 to 40 rev/min over 5 minutes and the amount of time it took for mice to fall off the bar was recorded.

References:

1. Fride E, Perchuk A, Hall FS, Uhl GR, Onaivi ES. Behavioral Methods in Cannabinoid Research. In: Onaivi ES (ed). *Marijuana and Cannabinoid Research: Methods and Protocols*. Humana Press: Totowa, NJ, 2006, pp 269-290.

2. Compton DR, Johnson MR, Melvin LS, Martin BR. Pharmacological profile of a series of bicyclic cannabinoid analogs: classification as cannabimimetic agents. *J Pharmacol Exp Ther* 1992; **260**(1)**:** 201-209.

3. Little PJ, Compton DR, Johnson MR, Melvin LS, Martin BR. Pharmacology and stereoselectivity of structurally novel cannabinoids in mice. *J Pharmacol Exp Ther* 1988; **247**(3)**:** 1046-1051.

Supplementary figure legends:

Figure Suppl-1 (related to Fig. 1): PPAR RNAscope ISH results - the same images as shown in Fig. 1 (A, B) under high magnification, illustrating that more DA neurons expressed PPARγ transcript (**B**) than DA neurons expressing PPARα transcript (**A**).

Figure Suppl-2: RNAscope ISH results indicating that PPARα transcripts were detected in a small subset of VGluT2-positive glutamate neurons and GAD1-positive GABA neurons in the VTA, NAc and amygdala.

Figure Suppl-3: RNAscope ISH results indicating that PPARγ transcripts were detected in a small subset of VGluT2-positive glutamate neurons and GAD1-positive GABA neurons in the VTA, NAc and amygdala.

Figure Suppl-4: PPARα/γ-immunostaining in the VTA, indicating that PPARα and PPARγ were detected in a subset of VGluT2-positive glutamate neurons and GAD67-positive GABA neurons.

Figure Suppl-5: PPARα/γ-immunostaining in the amygdala, indicating that PPARα and PPARγ were detected in a subset of VGluT2-positive glutamate neurons and GAD67-positive GABA neurons.

Figure Suppl-6: PPARα/γ-immunostaining in the NAc, indicating that PPARα and PPARγ were detected in astrocyte-like cells, not in GAD67-positive GABA neurons.

Figure Suppl-7. Δ^9-THC and/or PPAR antagonist-induced CPP or CPA. (**A**) The general CPP/CPA procedures. (**B/C**) Pretreatment with a PPARα (**B**) or PPARγ (**C**) antagonist failed to alter place aversions produced by Δ^9-THC. (**D**) The PPARα antagonist GW6471 failed to produce CPP or CPA, while (**E**) the PPARγ antagonist GW9662 produced CPA at 2 mg/kg. *p<0.05 relative to pre-conditioning. N = 8/group.

Figure Suppl-8 (related to Fig. 6). Δ^9-THC-induced tetrad effects in the presence or absence of the PPARα antagonist GW6471, demonstrating that pretreatment with

GW6471 enhanced THC-induced catalepsy (**A**), but failed to alter Δ^9-THC-induced analgesia (**B**), hypothermia (**C**), or immobility (**D**).

Figure Suppl-9 (related to Fig. 6): Δ^9-THC-induced tetrad effects in the presence or absence of the PPARγ antagonist GW9662, demonstrating that pretreatment with GW9662 enhanced THC-induced catalepsy (**A**), but failed to alter Δ^9-THC-induced analgesia (**B**), hypothermia (**C**), or immobility (**D**).
