# Supplementary material for: PPARα and PPARγ are expressed in midbrain dopamine neurons and modulate dopamine- and cannabinoid-mediated behavior in mice
Source: Mol Psychiatry. 2023 Jul 21;28(10):4203–14. doi: 10.1038/s41380-023-02182-0 (PMC10799974; doi:10.1038/s41380-023-02182-0)
Supplement: Supplementary file 2 [file 41380_2023_2182_MOESM12_ESM.docx]

**Supplementary Table 2.** Statistical analysis results by two-way RM ANOVAs over time and drug dose  

| **Figure** [**5**](https://bpspubs.onlinelibrary.wiley.com/doi/10.1111/bph.14958) (Locomotion) | **Drug treatment** **main effect** | **Time main effect** | **Treatment × time** **interaction** |
| --- | --- | --- | --- |
| Fig. 5A | *F*_2,21_ = 1.28; *P* >0.05 | *F*_11,231_ = 5.78; *P* <0.001 | *F*_22,231_ = 3.48; *P* <0.001 |
| Fig. 5B | *F*_2,21_ = 0.44; *P* >0.05 | *F*_11,231_ = 13.05; *P* <0.001 | *F*_22,231_ = 5.36; *P* <0.001 |
| Fig. 5C | *F*_2,21_ = 17.39; *P* <0.001 | *F*_11,231_ = 7.84; *P* <0.001 | *F*_22,231_ = 4.78; *P* <0.001 |
| Fig. 5D | *F*_2,21_ = 5.67; *P* <0.01 | *F*_11,231_ = 8.15; *P* <0.001 | *F*_22,231_ = 3.49; *P* <0.001 |
| Fig. 5E | *F*_3,28_ = 5.86; *P* < 0.01 | *F*_11,308_ = 15.37; *P* <0.001 | *F*_33,308_ = 4.65; *P* <0.001 |
| Fig. 5F | *F*_3,28_ = 3.67; *P* < 0.05 | *F*_11,308_ = 10.02; *P* <0.001 | *F*_33,308_ = 2.27; *P* <0.001 |
