# Supplementary material for: PPARα and PPARγ are expressed in midbrain dopamine neurons and modulate dopamine- and cannabinoid-mediated behavior in mice
Source: Mol Psychiatry. 2023 Jul 21;28(10):4203–14. doi: 10.1038/s41380-023-02182-0 (PMC10799974; doi:10.1038/s41380-023-02182-0)
Supplement: Supplementary file 3 [file 41380_2023_2182_MOESM13_ESM.docx]

**Supplementary Table 3.** Statistical analysis results by two-way RM ANOVAs over time and GW6471 dose

| **Figure S8** (Δ^9^-THC tetrad) | **Drug treatment** **main effect** | **Time main effect** | **Treatment × time** **interaction** |
| --- | --- | --- | --- |
| Fig. S8-A | *F*_2, 21_ = 0.81; *P* =0.458 | *F*_4, 84_ = 4.32; *P* <0.01 | *F*_8, 84_ = 1.24; *P* =0.287 |
| Fig. S8-B | *F*_2, 21_ = 7.78; *P* <0.01 | *F*_4, 84_ = 39.39; *P* <0.001 | *F*_8, 84_ = 3.18; *P* <0.01 |
| Fig. S8-C | *F*_2, 21_ = 1.27; *P* =0.303 | *F*_4, 84_ = 36.82; *P* <0.001 | *F*_8, 84_ = 0.84; *P* =0.568 |
| Fig. S8-D | *F*_2, 21_ = 1.16; *P* =0.334 | *F*_4, 84_ = 2.03; *P* =0.097 | *F*_8, 84_ = 0.62; *P* =0.762 |
| Fig. S8-E | *F*_2, 21_ = 0.25; *P* =0.781 | *F*_4, 84_ = 7.89; *P* <0.001 | *F*_8, 84_ = 0.55; *P* =0.814 |
| Fig. S8-F | *F*_2, 21_ = 2.89; *P* =0.078 | *F*_4, 84_ = 23.58; *P* <0.001 | *F*_8, 84_ = 0.84; *P* =0.574 |
| Fig. S8-G | *F*_2, 21_ = 2.58; *P* =0.099 | *F*_4, 84_ = 9.17; *P* <0.001 | *F*_8, 84_ = 1.43; *P* =0.196 |
| Fig. S8-H | *F*_2, 21_ = 0.25; *P* =0.784 | *F*_4, 84_ = 48.02; *P* <0.001 | *F*_8, 84_ = 0.16; *P* =0.995 |
| Fig. S8-I | *F*_2, 21_ = 0.25; *P* =0.782 | *F*_4, 84_ = 168.19; *P* <0.001 | *F*_8, 84_ = 0.32; *P* =0.955 |
| Fig. S8-J | *F*_2, 21_ = 0.69; *P* =0.512 | *F*_4, 84_ = 0.44; *P* =0.782 | *F*_8, 84_ = 0.54; *P* =0.826 |
| Fig. S8-K | *F*_2, 21_ = 0.67; *P* =0.522 | *F*_4, 84_ = 44.59; *P* <0.001 | *F*_8, 84_ = 0.82; *P* =0.590 |
| Fig. S8-L | *F*_2, 21_ = 1.28; *P* =0.300 | *F*_4, 84_ = 82.86; *P* <0.001 | *F*_8, 84_ = 1.13; *P* =0.349 |
